# Discovery of Laacher See eruption in speleothem record synchronizes Greenland and central European Late Glacial climate change

**DOI:** 10.1126/sciadv.adt4057

**Published:** 2025-01-15

**Authors:** Sophie F. Warken, Axel K. Schmitt, Denis Scholz, Andreas Hertwig, Michael Weber, Regina Mertz-Kraus, Frederick Reinig, Jan Esper, Michael Sigl

**Affiliations:** ^1^ Institute of Earth Sciences, Ruprecht-Karls-University Heidelberg, Im Neuenheimer Feld 234, 69120 Heidelberg, Germany.; ^2^ Institute of Environmental Physics, Ruprecht-Karls-University Heidelberg, Im Neuenheimer Feld 229, 69120 Heidelberg, Germany.; ^3^ John de Laeter Centre, Curtin University, 6845 Bentley, Australia.; ^4^ Institute for Geosciences, Johannes Gutenberg University Mainz, Johann-Joachim-Becher-Weg 21, D-55128 Mainz, Germany.; ^5^ Institute for Geography, Johannes Gutenberg University Mainz, Johann-Joachim-Becher-Weg 21, D-55128 Mainz, Germany.; ^6^ Department of Climate and Environmental Physics, University of Bern, 3012 Bern, Switzerland.; ^7^ Oeschger Centre for Climate Change Research, University of Bern, 3012 Bern, Switzerland.

## Abstract

To assess the impact of ongoing, historically unprecedented Arctic ice melting, precisely synchronized chronologies are indispensable for past analogs of abrupt climate change. Around 12,900 years before present (B.P.), the Atlantic-European realm experienced an abrupt relapse to near-glacial climate conditions attributed to Arctic meltwater fluxes, the Younger Dryas. However, it remained unclear how fast this climatic change propagated southward into Europe as terrestrial and ice-core chronologies are not sufficiently synchronized. Here, we use a volcanic sulfur spike identified in a speleothem from Germany to link the Laacher See eruption (LSE), a key chronostratigraphic marker in European terrestrial archives, to a previously unidentified sulfate spike in the Greenland ice-core record. The LSE, dated to 13,008 ± 8 years B.P._1950_, thus synchronizes radiometric and ice-core calendars back in time, which consistently demonstrates that the LSE predates the onset of the Younger Dryas cooling by about 150 years, both in Greenland and Europe.

## Teaser

A volcanic sulfur spike links the Laacher See eruption to Greenland ice cores and synchronizes the timelines to European records.

## INTRODUCTION

During the Last Glacial and deglaciation, global climate was characterized by extreme events recurring on a millennial scale (*1*). The most recent of these events is the Younger Dryas (YD) [approximately 12.9 thousand years (ka) before present (B.P.)] (*2*, *3*), when the Atlantic-European realm experienced an abrupt relapse to near-glacial climate conditions, most commonly attributed to North Atlantic meltwater fluxes and resulting variations in the strength of the Atlantic meridional overturning circulation (AMOC) (*4*, *5*). To assess the impact of ongoing, historically unprecedented Arctic ice melting and AMOC slowdown (*6*, *7*), knowledge of the exact causes, but particularly the timing and evolution of events during the onset of the YD, is of broad interest. However, it is still unclear whether the YD climatic changes occurred synchronously in the North Atlantic and European realm or whether they gradually propagated from Greenland to central Europe over several decades to centuries (*3*, *8*, *9*).

Tephra from the Laacher See eruption (LSE) is a crucial chronostratigraphic marker in Late Pleistocene central European paleoclimate records (*10*–*12*). Originating from the East Eifel volcanic field in Germany around 13 ka B.P., this event represents one of the largest known volcanic eruptions in central Europe during the Quaternary (*13*, *14*). Since the terrestrial and ice-core chronologies are not sufficiently synchronized, the temporal vicinity of the LSE to the Late Glacial YD cooling led to a debate about whether the LSE contributed to the inception of the YD in central Europe (*15*), as well as whether its timing allows to constrain how fast the climatic change associated with the YD propagated southward into central Europe (*8*, *9*, *16*).

Despite the long-recognized pivotal role of the LSE, robust constraints on its timing and impact have remained elusive. This is partially due to ambiguities about the magnitude of the eruption and its atmospheric sulfur release (*14*, *17*, *18*) but also since the accuracy of the date of the LSE remains a matter of debate. Previously dated in the varved Meerfelder Maar (MFM) record to 12,880 ± 40 varve years B.P. (*10*), an independent and precise age of this Late Glacial time marker could be inferred recently when radiocarbon (^14^C) measurements of subfossil trees buried by pyroclastic deposits of the LSE yielded an eruption age of 13,006 ± 9 calibrated years B.P. (cal. B.P.) (*9*). This date placed the timing of the LSE well before the onset of the YD and precluded a causal link between the eruption and the Late Glacial cooling in central Europe (*9*). However, this revised date has been questioned since ^14^C ages of the subfossil trees may be biased toward older ages because of the incorporation of ^14^C -free magmatic carbon dioxide (CO_2_) (*19*), a potential problem that has often been invoked in connection with the dating of past volcanic eruptions with ^14^C (*20*, *21*). Because of the absence of evidence for LSE cryptotephra in the polar ice strata (*22*), it remains unresolved whether any volcanic proxies from LSE are recorded in Greenland ice cores (*18*). This is aggravated by a lack of synchronization between varve (B.P._varve_) and ice-core layer counting (GICC05, B.P._GICC05_), ^14^C calibrated years (cal. B.P.), and the ^230^Th/U or other radiometric timescales (B.P._1950_) (*23*, *24*). Even though cosmogenic radionuclides (^10^Be and ^14^C) can provide age constraints across multiple archives (*23*), climate proxy records and volcanic event stratigraphies obtained from Greenland ice cores and other climate archives from central Europe have still not been unambiguously synchronized during the Late Glacial. The implications of an accurate age of the LSE are thus substantial and include the (mis)interpretation of regional climate records, the (mis)alignment of climatic shifts, and the exclusion or inclusion of the LSE as a possible driver of abrupt climate change (*18*, *19*). Identification of the LSE on the GICC05 and/or ^230^Th/U timescales would link the different chronologies and constrain the synchroneity of Late Glacial climate records in Europe and Greenland (*22*, *24*).

Speleothem δ^18^O values provide valuable high-resolution records of regional climate and temperature that are coherent across central Europe and the North Atlantic realm (*3*, *25*, *26*). In addition, speleothems have been shown to preserve signatures of volcanic eruptions via enhanced sulfur contents and/or other ash-leached trace element abundances (*27*–*30*). The ^230^Th/U dating method applied to speleothems yields precise absolute chronologies for the climate proxy time series and volcanic eruptions recorded in speleothems. Here, we show geochemical evidence of the LSE event recorded in a speleothem from Germany from a cave located below the main tephra fallout fan that allows us to align the key chronologies of Greenland and European climate records. Statistical analysis of the timing of the LSE in different climate archives permits us to identify the LSE in Greenland ice cores and synchronize the speleothem ^230^Th/U chronology with the Greenland GICC05 timescale. This yields an updated, absolute, and accurate age of the LSE of 13,008 ± 8 years B.P._1950_, which corresponds to a previously identified but unassigned bipolar ice-core volcanic sulfate peak at 12,994 years B.P._GICC05_. Our study unequivocally confirms that the LSE predates the Late Glacial YD cold event in central Europe to an extent that precludes a causal relationship. In addition, our synchronization with the ice-core chronology demonstrates that the onset of the YD is indistinguishable from the transition from Greenland interstadial 1a (GI-1a) to Greenland stadial 1 (GS-1), suggesting an immediate coupling of central European and Arctic climate.

## RESULTS

### Cave setting and LSE fingerprint

**Fig. 1.**
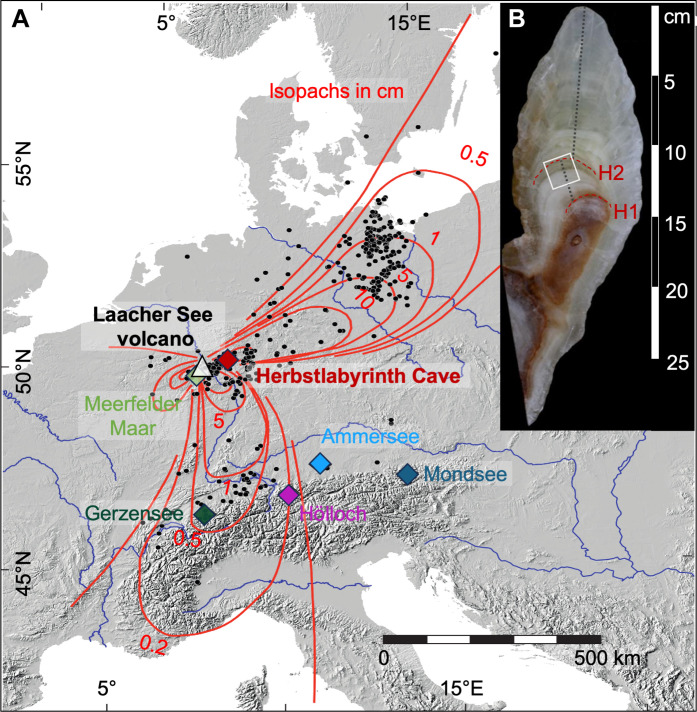
Location and sample overview. (**A**) Location of Laacher See volcano, Herbstlabyrinth Cave, and other sites discussed in the text in central Europe. Also shown are the main tephra lobes of the LSE. Black dots indicate the locations of known Laacher See tephra deposits (*71*–*75*), and Laacher See tephra thicknesses (isopachs) are indicated by red lines (*14*). The map uses Europe Albers equal area conic projection. (**B**) Scan of stalagmite HLK2 indicating the growth axis (dotted black line). The Late Glacial growth phase between growth stops H1 and H2 is highlighted by the white frame.

**Fig. 2.**
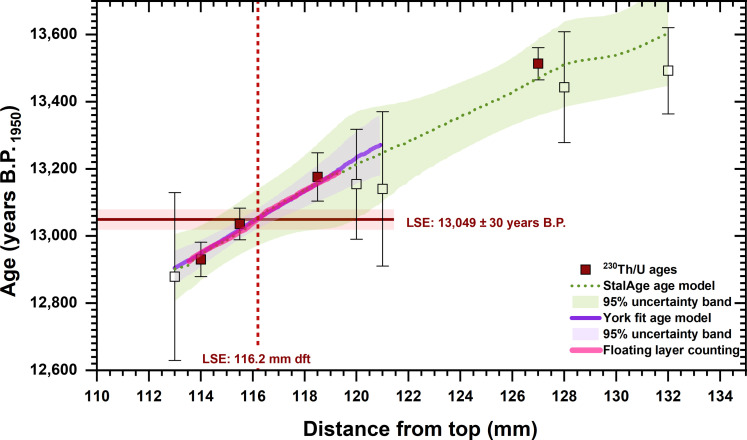
^230^Th/U dating results (symbols) and chronology of HLK2. Open symbols indicate the ages by Mischel *et al.* (*32*), and filled symbols outline the data from this study. The agreement of fluorescence layer counting (pink) and linear York fit (purple) demonstrates linear growth in the LSE search section (~119- to 114-mm dft). The York fit yields an age of 13,049 ± 30 years B.P. for the LSE anomaly identified at 116.2-mm dft. The green dashed line shows the StalAge chronology (*76*) constructed for the whole Late Glacial growth phase of HLK2.

Speleothem HLK2 has been collected from Herbstlabyrinth Cave, western Germany, which is situated ~70 km northeast of the Laacher See (Fig. 1) and well within the main northeast fallout lobe of the eruption (*14*, *31*). A refined ^230^Th/U age model of this Late Glacial speleothem record (*32*) enabled high-resolution investigation of the speleothem geochemistry between approximately 13,600 and 12,750 years B.P._1950_, i.e., along the proposed LSE age estimates (Fig. 2). The LSE was geochemically fingerprinted with high-resolution methods, including secondary ionization mass spectrometry (SIMS) and laser ablation inductively coupled plasma mass spectrometry (LA-ICP-MS) (see Materials and Methods and figs. S1 to S3).

**Fig. 3.**
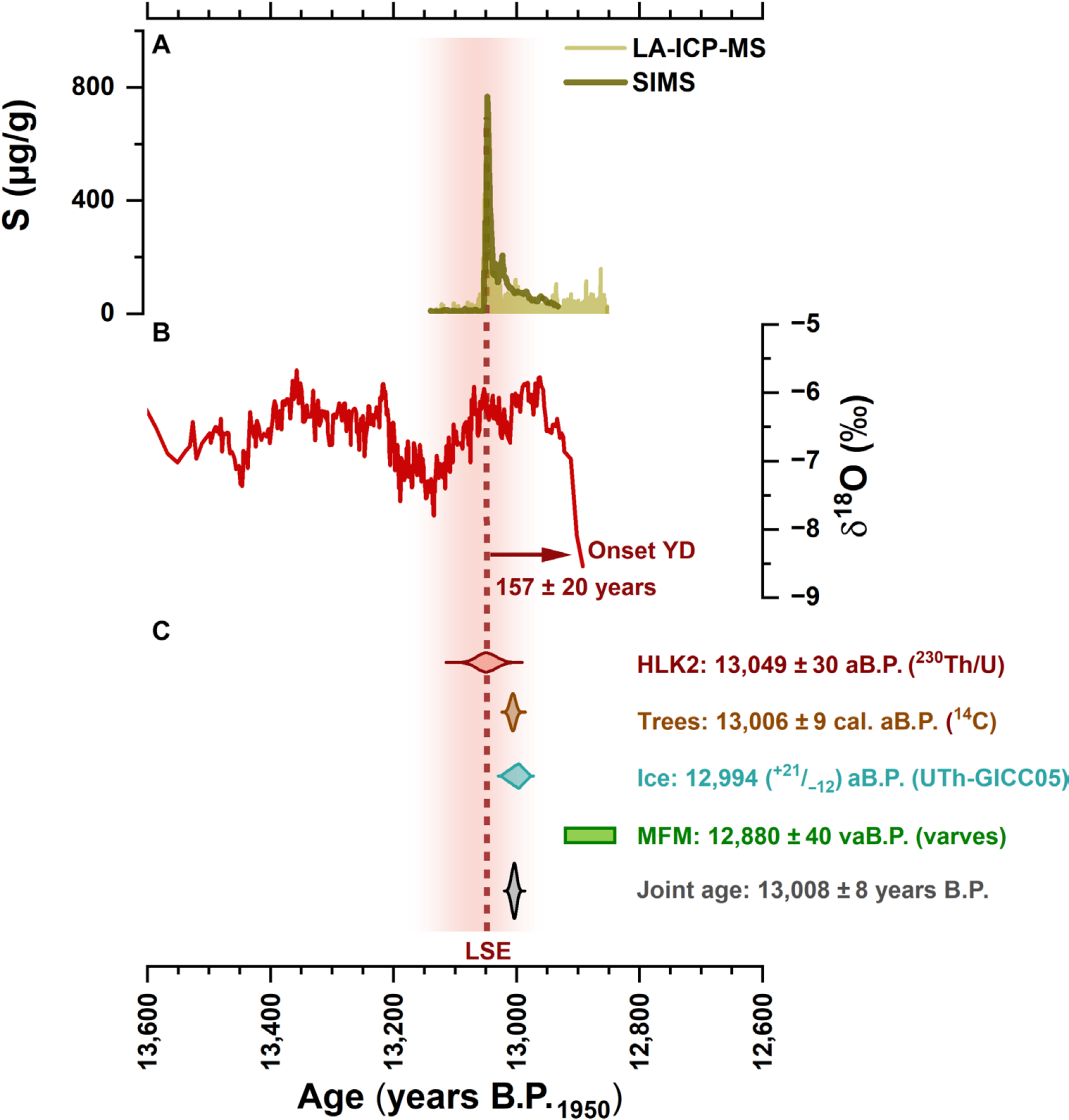
Speleothem HLK2 geochemistry and LSE signal. From top to bottom: (**A**) sulfur concentrations obtained by SIMS (dark yellow) and LA-ICP-MS (bright yellow). (**B**) Speleothem calcite δ^18^O profile. The vertical dashed line indicates the timing of the LSE relative to the δ^18^O record. The data shown in both panels can be found in tables S2 to S4. (**C**) Age distribution of the independent ^230^Th/U data from HLK2 (red) compared to other LSE dates (see text for more details). The gray symbol shows the overlapping, joint age distribution of speleothem, ice, and trees, excluding the MFM date.

In the stalagmite, the LSE is clearly detectable by a prominent spike in sulfur (Fig. 3A) and other ash-leached trace element abundances such as Ba, U, or Na (figs. S3 and S4). The geochemical evidence is consistent with the substantial sulfur emissions by the LSE estimated at 3.5 to 150 Tg (*14*, *17*), as well as the alkali-rich phonolitic composition of LSE tephra (*33*) (more details in Supplementary Text). Strongly increased fluorescence intensity further indicates an enhanced flux of degraded organic matter after the eruption (figs. S2 and S4). The duration of the volcanogenic anomaly in HLK2 agrees well with the previously reported immediate impact of the LSE on vegetation in the proximal regions that lasted over ~20 years after the eruption (*34*).

Beyond this abrupt and local influence following the eruption, neither annually resolved δ^18^O data (Fig. 3B), lamina thickness, nor trace element abundances (fig. S4) provide any evidence of a long-term environmental impact, arguing for a limited climatic significance of the LSE. However, a prominent drop in the HLK2 δ^18^O values can be observed 157 ± 20 years after the LSE, indicating the onset of the YD (Fig. 3B). The δ^18^O decrease by several per mil occurs coeval with changes in moisture-sensitive elemental proxies in HLK2, suggesting that the YD cooling in the North Atlantic realm was associated with a change of local hydroclimatic conditions (fig. S4; more details in the Supplementary Materials).

On the basis of the ^230^Th/U age model of speleothem HLK2 (Fig. 2), the LSE spike has an age of 13,049 ± 30 years B.P._1950_ (2σ) (Fig. 3C). This age is significantly older than the varve counting age from MFM [12,880 ± 40 years B.P._varve_ (*10*); Fig. 3C] or the ^14^C-based age range from Lake Soppensee (12,735 to 12,871 cal. years B.P.) (*35*). However, it agrees within uncertainty with the ^14^C age of the LSE tephra in Lake Lucerne [12,972 cal. years B.P. (*36*)] or in the stacked Gerzensee δ^18^O record [13,034 years B.P._GICC05_ (*12*)] obtained via event-stratigraphical correlation with Greenland ice-core δ^18^O values (*37*) on the GICC05 timescale (*1*). Our data also support the earlier findings in (*9*) that the previously reported age for the LSE is too young and clearly rule out that the ^14^C wiggle matching age (13,006 ± 9 cal. B.P.) is biased by addition of magmatic CO_2_.

## DISCUSSION

### Linking the LSE in speleothem and Greenland ice-core records

**Fig. 4.**
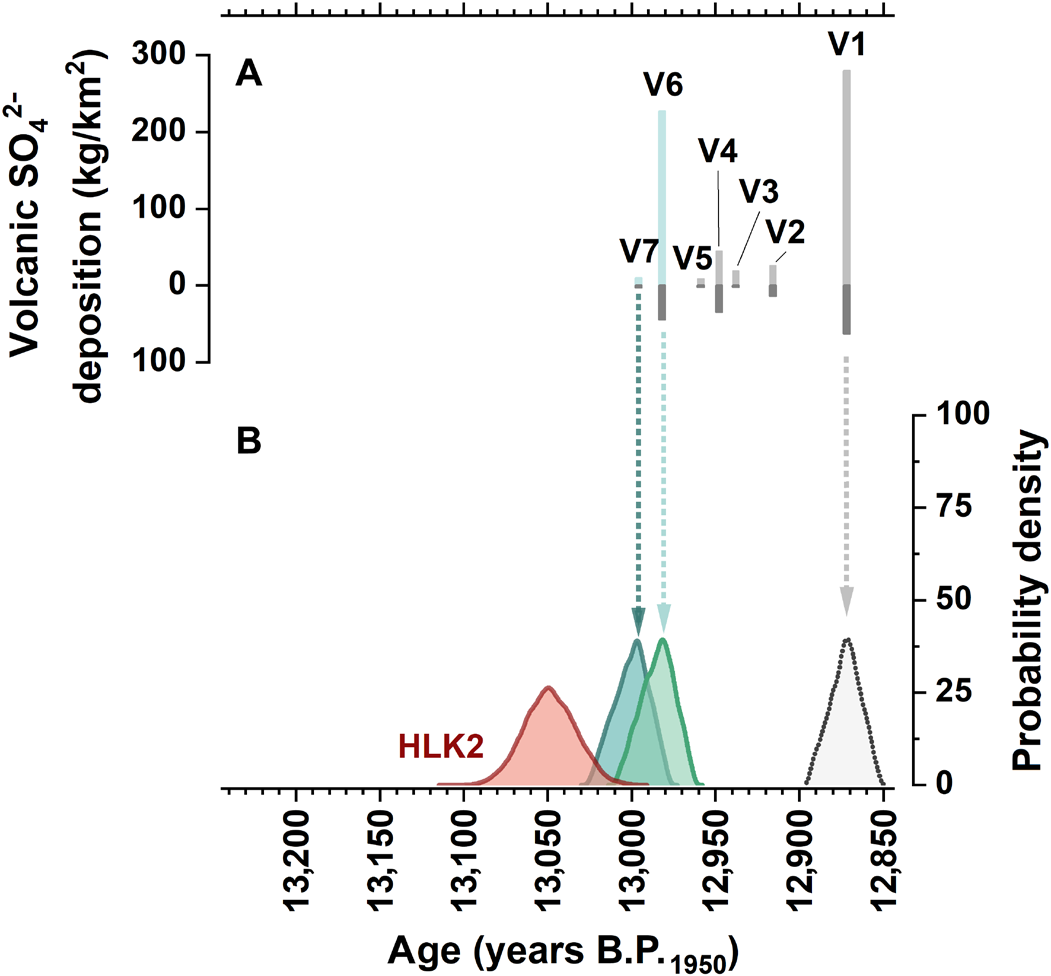
Statistical analysis of LSE candidate events in ice cores. (**A**) Volcanic sulfate deposition in Greenland (upper scale) and Antarctica (lower scale), modified after the compilation by Abbott *et al.* (*18*). Only candidate events that meet the prerequisites of a reconstructed volcanic stratospheric sulfur injection >3.5 gT and an interhemispheric AR > 0.3, indicating a Northern Hemisphere eruption, are shown (compare table S5). (**B**) LSE age distribution density plots from speleothem HLK2 (this study, red) compared to the age distributions of candidate events V1, V6, and V7 based on the U-Th–corrected GICC05 chronology (*23*). The plot for V5 is shown in fig. S5.

The high-precision independent speleothem ^230^Th/U age opens a unique pathway to investigate the accurate timing of the LSE not only in conjunction with other age determinations but in particular toward a potential signature of the LSE in the Greenland ice-core sulfate records (*18*). The chemical signature of the LSE remains elusive in Greenland despite sampling several ice cores over depth ranges that encompass best age estimates (*9*, *18*, *22*, *38*). High-resolution search efforts did not locate tephra from the compositionally distinctive LSE in chemical profiles from Greenland ice cores (*22*). Since other key tephra from explosive eruptions from central and southern Europe (e.g., Vesuvius in 79 CE) is also absent in Greenland, external mechanisms, such as predominant wind patterns as the jet stream, may have acted as barriers inhibiting the direct northwestward transport of tephra (*22*, *39*, *40*). Linking a sulfate signal in the ice cores to the LSE is additionally hindered by the high uncertainty regarding the expected atmospheric sulfate burden, as estimates of sulfur emitted during the eruption range between 3.5 and 150 Tg (*14*). Consequently, within the LSE search window from approximately 13,050 to 12,870 years B.P._GICC05_, seven volcanic events in the ice-core record meet the LSE sulfur emission constraints, i.e., a sufficiently high reconstructed volcanic stratospheric sulfur injection and an interhemispheric sulfate deposition asymmetry ratio (AR) typical for extratropical eruptions in the Northern Hemisphere (Fig. 4A and table S5).

A Monte Carlo simulation of the LSE ages obtained from the ^230^Th/U-dated HLK2 record and the U-Th–corrected GICC05 ice-core chronology (*23*) allows us to quantify the probability of the seven volcanic events as candidates for the LSE (fig. S5). The resulting distribution of chronologies is a measure for the probability that the corresponding events are coeval with the LSE in the HLK2 record and thus represent the LSE. The particularly strong event at 12,870 years B.P._GICC05_ (V1), which has been repeatedly brought up in the discussion because of its magnitude and timing close to GS-1 (*15*, *19*), can be confidently excluded as a candidate for the LSE (*P* < 0.0001) because its timing is significantly younger than in the speleothem (Fig. 4B and fig. S5A). The same applies to events V5–V2, which are younger than 12,957 years B.P._GICC05_ (fig. S5A). For V6 (12,980 years B.P._GICC05_), a very small fraction of 1% of all simulated ages overlaps with the age distribution of the speleothem LSE spike (Fig. 4B and fig. S5C). At the 99% confidence level, this event can also be excluded to represent the LSE. For V7 (12,994 years B.P._GICC05_; Fig. 4B); however, 12% of the simulated ages overlap with the LSE spike in the speleothem, and thus, this volcanic event represents the only likely candidate for the LSE.

The annually resolved HLK2 δ^18^O data further allow to determine the correlation with the Greenland ice-core δ^18^O values. The synchroneity of centennial- to millennial-scale transitions in δ^18^O records from central Europe and ice cores in Greenland has been demonstrated in various modern and (Late) Glacial records (*3*, *12*, *26*, *41*, *42*), including the Herbstlabyrinth Cave speleothems (*32*, *43*). The recently developed high-resolution HLK2 δ^18^O record also outlines this close relationship with a correlation to North Greenland Ice Core Project (NGRIP) δ^18^O values of *r* = 0.39 (^0.28^/_0.79_) (mean value and 95% confidence interval), considering the dating uncertainties of both records (fig. S5). Notably, the correlation shows a maximum [*r* = 0.56 (^0.31^/_0.84_)] for those simulations when the LSE age in the speleothem is coeval with the sulfur spike of V7 (fig. S5H). For event V6, the correlation is close to the maximum [*r* = 0.55 (^0.27^/_0.86_)] but shows a temporal lag of 16 ± 2 years (fig. S5G). For all other events, the correlation is significantly lower or absent (fig. S5, E and F).

Given the expected synchronous response of the δ^18^O signals in Greenland and central Europe to changes in common moisture sources and temperature, this correlation analysis strongly underscores our aforementioned findings that the statistically by far most likely candidate for the LSE in the ice-core record is the event V7 at 12,994 years B.P._GICC05_. Since V7 is a relatively small sulfate peak, we argue that the LSE represents a medium- rather than high-sulfur-emission scenario (*18*), in line with the lack of success in emission-based search efforts to identify the LSE in the ice-core record (*22*), as well as the limited climatic consequences of the LSE recorded in HLK2 and lake sediment records.

### Joint LSE age synchronizes central European and Greenland chronologies

Our age distribution and correlation analyses provide statistical evidence to identify a single candidate event for the LSE in the ice-core record. Within the comparably small 95% confidence intervals (^−12^/_+21_) of the U-Th–corrected GICC05 timescale (*23*), the V7 candidate event at 12,994 years B.P._GICC05_ is the only event significantly overlapping with our ^230^Th/U age of the LSE (13,049 ± 30 years B.P._1950_). The overlap of our ^230^Th/U age with the age distribution for event V7, as well as with the precise radiocarbon age in (*9*), enables us to calculate a joint age distribution and refine the age of the LSE to 13,008 ± 8 years B.P._1950_ (Fig. 3C).

As a chronostratigraphic marker, the LSE tephra links various proxy records in central Europe, and the age determined in (*9*) demonstrated that, as a consequence, the YD cooling must have occurred synchronously in central Europe. However, so far, a comprehensive synchronization, including the Greenland ice-core records, has not been possible. Around 13,000 years B.P._1950_, a synchronization of the GICC05 chronology with the ^230^Th/U and radiocarbon timescales via the LSE would thus require a shift of GICC05 by +14 years on average and reduce the relative chronological uncertainty in this section to ~0.6 per mil (‰) (2σ). With our statistical approach, the LSE thus becomes a precise and accurate chronostratigraphic marker for the transition from the Late Pleistocene into the Holocene also in the ice-core record.

**Fig. 5.**
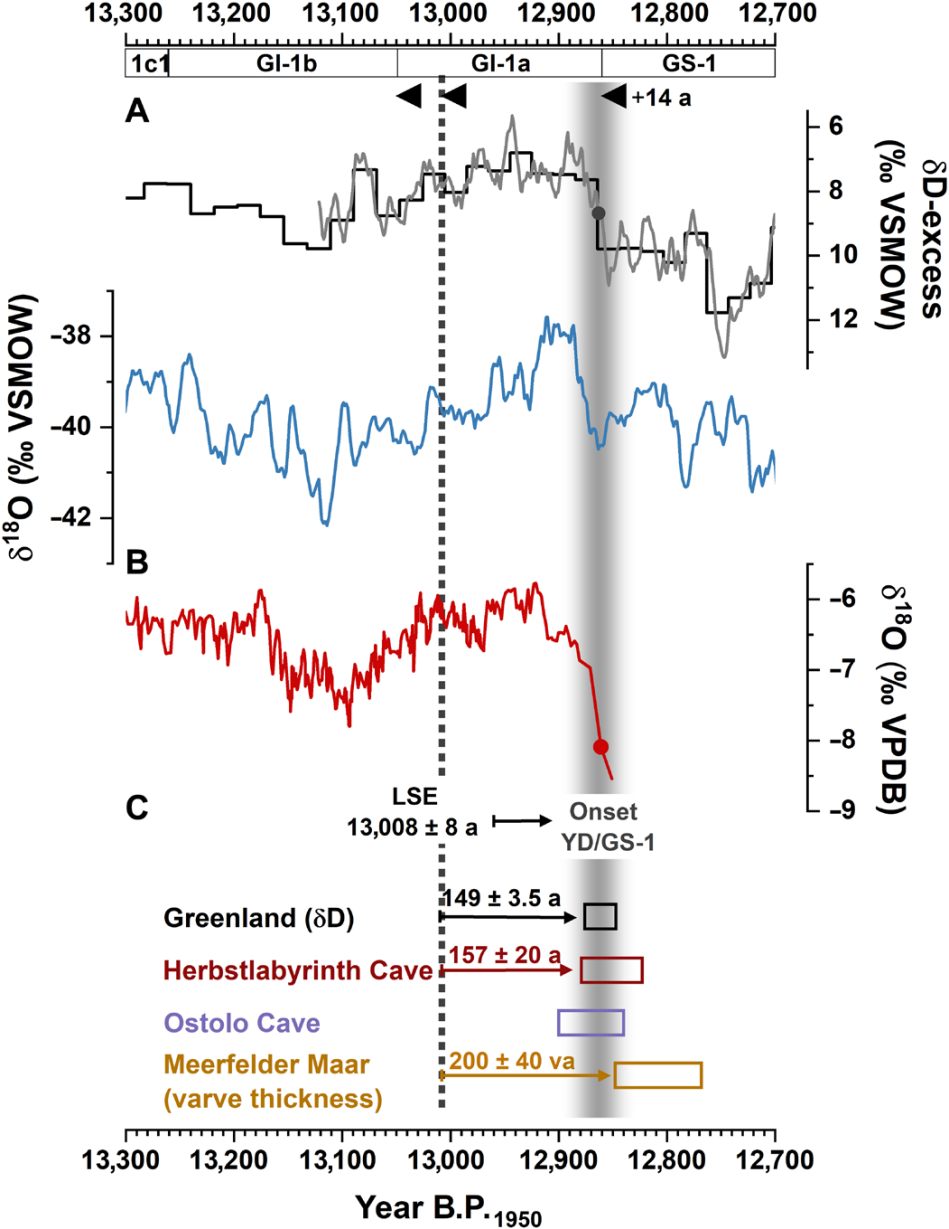
Comparison of Greenland and European records. From top to bottom: (**A**) 10-year running mean of the Greenland NGRIP ice-core δ^18^O (*37*) and δD-excess (*44*) data on the revised U-Th-GICC05 age scale (*23*) but shifted by +14 years according to the updated LSE date. VSMOW, Vienna standard mean ocean water. (**B**) Composite δ^18^O record from Herbstlabyrinth Cave (this study). VPDB, Vienna Pee Dee belemnite. (**C**) Comparison of the timing of the onset of the YD/GS-1 after the LSE in Atlantic-European including Greenland and HLK2 [(A) and (B)], as well as Ostolo Cave (northern Spain) (*42*) and the varve counted chronologies from MFM (*10*). Colored boxes indicate the timing and uncertainty range of the onset of the YD/GS-1 in the individual records (see text for more details). The vertical dashed line indicates the age of the LSE, and the vertical shading indicates the timing of the onset of the YD/GS-1 cooling in the Atlantic-European realm.

The identification of the LSE in the ice-core record now permits a direct comparison of transatlantic climate records from high and mid-latitudes. Consequently, this allows a profound assessment of Late Glacial climate relationships and abrupt transitions, which has previously been rather speculative, given the prevailing age uncertainties. One major consequence of linking the chronologies via the LSE is that the onset of the central European YD cooling is synchronous with the timing of the transition from GI-1a to GS-1. In line with the findings in (*9*), the initiation of the YD as the onset of the pronounced cooling occurs at least 157 ± 20 years after the LSE in the HLK2 δ^18^O record, i.e., not before 12,851 ± 28 years B.P._1950_ (Fig. 5). This date agrees well with the previously determined timing for the abrupt YD cooling in the North Atlantic region recorded in a speleothem δ^18^O record from Ostolo Cave, Spain (12,870 ± 30 years B.P._1950_) (*3*) (Fig. 5).

**Fig. 6.**
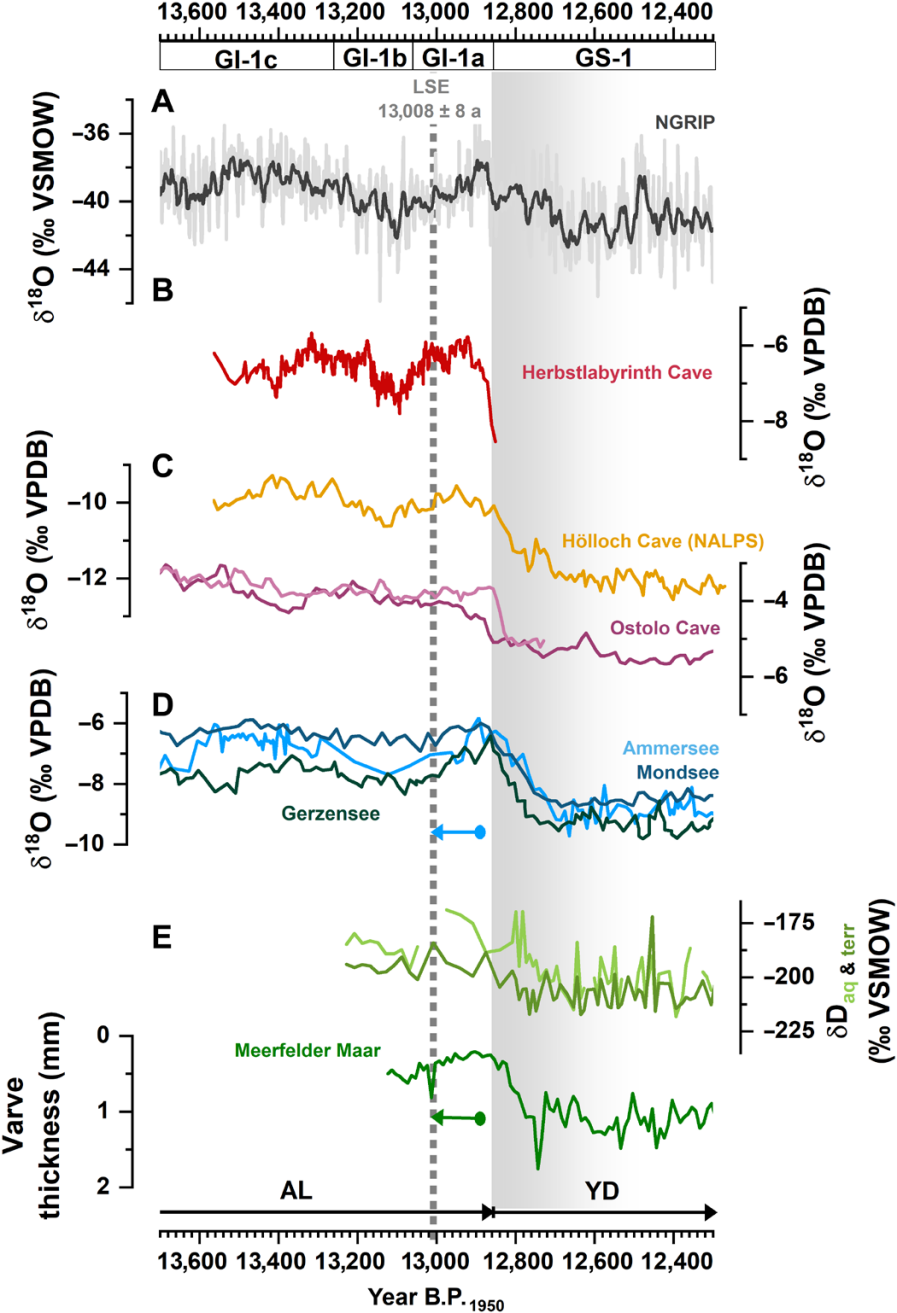
Comparison of Greenland and central European Late Glacial climate records. (**A**) Ten-year running mean of the Greenland NGRIP ice-core δ^18^O (*37*) on the revised U-Th-GICC05 age scale (*23*) but shifted by +14 years according to the updated LSE date. (**B**) Composite δ^18^O record from Herbstlabyrinth Cave (this study). (**C**) European stalagmite δ^18^O records from Hölloch Cave (northern Alps) (*52*) and Ostolo Cave (northern Spain) (*42*); (**D**) δ^18^O records from lakes Ammersee (*45*), Mondsee (*46*), and Gerzensee (*12*). (**E**) *n*-Alkane δD data (*16*) and varve thickness data (*10*) from MFM. The vertical dashed line indicates the timing of the LSE. All records [besides (C)] are aligned according to the age difference between the previous LSE dates in each record to 13,008 years B.P._1950_ (this study); horizontal arrows indicate the extent of the shift. The vertical gray shading shows the timing of the YD/GS-1 cooling.

The onset of GS-1 on the GICC05 timescale (not synchronized to the LSE) occurs at 12,846 (^−13^/_+16_) years B.P._1950_. Notably, the relative timing between the LSE and the onset of the YD in HLK2 is indistinguishable from the number of 149 ± 3.5 annual layers in NGRIP counted after V7 until the transition from GI-1a to GS-1 on the GICC05 timescale (*44*). When the ^230^Th/U and GICC05 chronologies are linked via the LSE and the timing of GS-1 would also be shifted by +14 years, the onset of GS-1 would be dated to approximately 12,860 years B.P._1950_, in even better agreement with the speleothem δ^18^O records. This demonstrates that the onset of the YD in central Europe in the speleothem records is within two to three decades indistinguishable from the onset of GS-1 in Greenland (Fig. 6). The updated LSE age also allows the synchronization of European lake chronologies with both the ice-core and speleothem records.

When the MFM varve chronology is adjusted with the updated LSE age, the onset of the YD identified in a decrease in varve thickness occurs at 12,808 ± 40 years B.P._1950_ (*10*). Within uncertainty, this date overlaps with the onset of the YD/GS-1 in the δ^18^O records from Greenland, Spain, and Germany (Fig. 5). In addition, the MFM leaf wax δD isotope records, interpreted as proxies of atmospheric and hydrological processes (*16*), as well as the δ^18^O data from the lakes Ammersee (*45*), Mondsee (*46*), and Gerzensee (*12*), show a consistent, synchronous pattern when synchronizing their chronologies to the updated LSE age (Fig. 6).

Within uncertainty, the Atlantic-European ice, lake, and speleothem records demonstrate a regionally coherent onset of the YD/GS-1 that is synchronous within a few decades and, hence, much smaller than the previously hypothesized time lag of up to 200 years (*16*). This is fully consistent with the previous results in (*9*). This also suggests that potentially remaining discrepancies or lags in individual records may be rather attributed to chronological uncertainties or proxy-specific, i.e., delayed or smoothed, responses.

### Consequences of European and Greenland synchroneity

The identification of the LSE in the ice-core records allows the alignment of Late Glacial European and Greenland chronologies and constrains the synchroneity of speleothem and ice-core δ^18^O records. This demonstrates that GS-1 and the YD, as inferred from Atlantic and European records, are identical and occurred ~150 to 160 years after the LSE. Moreover, our results not only preclude a direct link between the LSE and the YD/GS-1 cooling (*15*) but also propose a limited climatic significance of the eruption itself.

The origin of the YD/GS-1 cooling has been attributed to a substantial reduction in North AMOC because of sudden meltwater release from the Laurentide Ice Sheet in North America (*4*, *5*). Our results show that not only the cooling but also the related atmospheric and hydroclimatic response, as indicated by the various δ^18^O records, propagated rapidly from the North Atlantic realm into central Europe. This direct and dynamic coupling of mid- to high-latitude climate is not only opposed to a time-transgressive spread of the YD/GS-1 cooling from Greenland to central Europe (*3*, *8*, *9*, *16*) but also particularly relevant in the context of recent anthropogenic warming, increasing meltwater discharge to the North Atlantic and the associated AMOC weakening (*6*, *7*, *47*). This demonstrates the fundamental role of precise, accurate, and synchronized chronologies, in particular for inferences on the timing of climatic transitions and sequence of events.

## MATERIALS AND METHODS

### Site and sample description

The Herbstlabyrinth Cave system is located 435 m above sea level in the Rhenish Massif (Fig. 1) and is developed in Devonian limestone (*32*, *43*). The cave system is covered by 60-cm-thick soil and a patchy vegetation of meadow and deciduous forest (*43*). Stalagmite HLK2 (Fig. 1) is 15 cm long and was collected from a small chamber of Herbstlabyrinth Cave ~30 m below the surface (*32*). Details about petrography as well as stable isotope and trace elemental composition can be found in (*32*, *48*). Stalagmite HLK2 was actively growing at the time of collection. From September 2010 onward, a 5-year-long monitoring program was performed in and above the cave system, with drip water sampling and logger sites located in close proximity to this stalagmite (*43*).

Herbstlabyrinth δ^18^O values are representative of regional rainfall δ^18^O and thus closely linked to the North Atlantic moisture source and regional temperature from interannual to millennial timescales (*32*, *41*, *43*, *48*), which is not only common for German caves (*49*, *50*) but also across central and western Europe (*42*, *51*, *52*). Cave monitoring further shows that the transport of organic matter in Herbstlabyrinth drip waters occurs on a seasonal scale (*53*, *54*), consistent with an influx and subsequent incorporation of colloidal or particulate components into the speleothems in response to enhanced infiltration (*43*).

### ^230^Th/U dating

To precisely constrain the Late Glacial growth phase of speleothem HLK2 (*32*), additional samples for U-series dating were cut using a diamond-wire saw in chunks of ~00 to 270 mg. Chemical separation of U and Th was performed under clean laboratory conditions at the Institute for Geosciences, Johannes Gutenberg University (JGU) Mainz, using trace metal grade acids. Before dissolution, samples were leached in weak HNO_3_. After dissolution, a previously calibrated ^229^Th-^233^U-^236^U spike solution was added, and sample processing was performed using ion exchange columns filled with 1.5 ml of Bio-Rad AG 1-X8 anion exchange resin (*55*). Final fractions of U and Th were dissolved in 0.8 M HNO_3_ with 1‰ HF. Mass spectrometric analyses were performed using a Neptune Plus MC-ICP-MS system and an ESI Apex Omega HF desolvator with a sample uptake rate of ~100 μl/min. U and Th are analyzed separately in a standard-sample bracketing procedure (using CRM 112-A for U samples and an in-house Th standard including ^229^Th, ^230^Th, and ^232^Th for Th samples) to correct for mass fractionation and Faraday cup (FC) to ion counter gain. The abundance sensitivity is determined in separate analyses to correct for potential tails on isotopes with low abundance (i.e., ^230^Th and ^234^U). Detrital contamination of speleothem samples can become substantial if (^230^Th/^232^Th) < 200. Here, we follow the conventional approach assuming an upper continental crust ^232^Th/^238^U weight ratio of 3.8 (*56*) with an uncertainty of 50% (*57*) and ^230^Th, ^234^U, and ^238^U in secular equilibrium for the detrital material to account for initial Th.

### Elemental analysis

Analyses on three pieces of HLK2 were performed in line-scan mode at the Institute for Geosciences, JGU, Mainz, Germany, using an ESI NWR193 ArF excimer laser ablation system equipped with the TwoVol^2^ ablation cell, operating at 193-nm wavelength, coupled to an Agilent 7700x quadrupole ICP-MS. Before each line scan, surfaces were preablated to prevent potential surface contamination. For analyses, line scans were carried out at scan speeds of 10 μm/s (HLK2_1) and 2 μm/s (HLK2_2 and HLK2_3) using a rectangular beam of 130 μm by 50 μm (beam for preablation was 150 μm by 50 μm). The laser repetition rate was 10 Hz, and the laser energy on the samples was about 5 J/cm^2^. Background intensities were measured for 15 s. The monitored isotopes were ^23^Na, ^25^Mg, ^31^P, ^32^S, ^34^S, ^43^Ca, ^88^Sr, ^138^Ba, and ^238^U. The total list of monitored isotopes included ^7^Li, ^11^B, ^23^Na, ^25^Mg, ^27^Al, ^31^P, ^32^S, ^34^S, ^39^K, ^43^Ca, ^45^Sc, ^49^Ti, ^53^Cr, ^55^Mn, ^56^Fe, ^59^Co, ^60^Ni, ^63^Cu, ^66^Zn, ^85^Rb, ^88^Sr, ^89^Y, ^90^Zr, ^93^Nb, ^95^Mo, ^111^Cd, ^133^Cs, ^138^Ba, ^139^La, ^140^Ce, ^141^Pr, ^146^Nd, ^147^Sm, ^153^Eu, ^157^Gd, ^159^Tb, ^163^Dy, ^165^Ho, ^167^Er, ^169^Tm, ^173^Yb, ^175^Lu, ^181^Ta, ^208^Pb, ^232^Th, and ^238^U. The synthetic glass NIST SRM 610 was used to calibrate element concentrations by applying the preferred values available from the GeoReM database [http://georem.mpch-mainz.gwdg.de/; compare also Jochum *et al.* (*58*, *59*)]. Quality control materials (QCMs) (USGS MACS-3, USGS BCR-2G, NIST SRM 612, and KCSp-1-NP) were used to monitor the accuracy and precision of the LA-ICP-MS analysis and calibration strategy. Signals of all measurements were monitored in time-resolved mode and processed using the data reduction software iolite4 (*60*).^43^Ca was used as an internal standard applying for the samples a Ca concentration of 400,000 μg/g and the values reported in the GeoReM database for the calibration material and all QCMs but KCSp-1-NP. For KCSp-1-NP, the Ca concentration reported in (*61*) was used. Averaged element concentrations of repeated measurements (*n* = 12) of the QCMs agreed mostly within 10% with the reference values (i.e., the preferred values of the GeoReM database for NIST SRM 612 and USGS BCR-2G), the preliminary reference values for MACS-3 (*62*), and the informative values for KCSp-1NP given in (*61*) and had a relative SD of <5%. The results of the three line scans are given in table S2.

### Ion microprobe δ^18^O and S analyses

Guided by the low-resolution IRMS δ^18^O data and age-depth model for stalagmite HLK2 (*32*), two ~10-mm-sized pieces were cut using a low-speed diamond saw and placed on adhesive tape along with fragments of calcite reference material CCmb and NIST SRM 610. After embedding the speleothem fragments and reference materials in two epoxy disks (25.4 mm in diameter and ~5 mm thick; SIMS mounts 1 and 2), surfaces were ground and polished using diamond suspension. The mounts were then ultrasonically cleaned with deionized water and methanol. A ~50-nm conductive surface layer was applied using a QUORUM Q15OT ES high-vacuum sputter coater with a high-purity Au target before SIMS analysis. Oxygen isotope analyses on the CAMECA IMS 1280-HR ion microprobe at Heidelberg University were conducted in three analytical sessions (S1, S2, S3; see table S2a), which followed a protocol modified from Treble *et al.* (*63*). A 0.8- to 1.8-nA Cs^+^ beam impacting at a total energy of 20 keV was initially rastered over an area of ~15 μm by 15 μm over 20 s with a normal incidence electron gun providing charge compensation. After automatic centering of the secondary beam in the field aperture, ions with intensities of ~2 × 10^6^ to 6 × 10^6^ counts per second for ^18^O^−^ were then collected simultaneously in FCs with 10^10^- and 10^12^ -ohm feedback resistors for ^16^O^−^ and ^18^O^−^, respectively. The mass spectrometer was set to a mass resolving power (at 10% of the peak intensity) of ~2400 (slit 1 of the multicollection array). For data acquisition, a raster [5 μm by 5 μm (S2) or 10 μm by 10 μm (S1 and S3)] was used and counts were averaged over 15 cycles of 4-s integration time each (S2 and S3) or 20 cycles of 4 s integration time (S1). Raw counts were corrected for detector yields previously calibrated by applying reference voltages to the electrometers and for the detector baseline measured during the 20-s presputtering interval before each analysis. Detector baselines were then either averaged in a sliding window of five consecutive analyses (S1) or fitted using a polynomial function (S2 and S3) for subtracting the FC detector baselines. Instrumental mass fractionation α was corrected for by bracketing analyses of the in-house calcite reference material CCmb [28.81 ± 0.15‰, 2 s, Vienna standard mean ocean water, cross calibrated by SIMS using calcite reference material S0160 (*64*)]. The resulting α value was ~0.995, and the typical repeatability was 0.2 to 0.3‰ (2 SDs). This uncertainty is adopted as the external error for an individual analysis spot. Composite δ^18^O records of IRMS and SIMS data were constructed using the software ISCAM (*65*) (fig. S6).

To determine S abundances, spot analyses were subsequently performed along a ~0.4-mm-long section previously identified by δ^18^O analysis to cover the expected LSE age using the CAMECA IMS 1280-HR at Heidelberg University. A ~1-nA Cs^+^ beam at 20-keV impact energy was rastered over an area of 15 μm by 15 μm for 180 s to presputter the surface and remove potential contaminants; charge compensation was achieved via a normal incidence electron beam. The presputter duration was determined by monitoring the decay of the initially elevated ^32^S^−^ signal to a steady value. After the presputtering, the raster size was decreased to 10 μm by 10 μm to sequentially collect secondary ions of ^28^Si^−^, ^12^CO^−^, ^31^P^−^, ^32^S^−^, ^16^O_2_^−^, and ^35^Cl^−^ using the axial electron multiplier except for ^16^O_2_^−^ for which the axial FC2 detector was used. After dead-time and base-level corrections, the secondary ion intensities were normalized to ^16^O_2_^−^ and abundances were estimated from a relative sensitivity correction on the basis of analysis of NIST SRM 610 and 612 glasses (*59*) and in-house calcite reference material CCmb (38.2 μg/g S, by bulk acid digestion ICPMS, Agilent 8800 QQQ, ETH Zürich). A notable matrix effect between calcite and glass was found with calculated abundances differing by a factor of ~3. Because of the matrix effect and a wide range of S abundances reported for NIST SRM 610 and 612 glasses, we prefer the values calibrated on CCmb, emphasizing that only the relative abundances are important to identify LSE in the speleothem. Using the repeatability of CCmb replicate analyses as an estimate for the analytical uncertainty, S abundances are stated with a relative uncertainty of 14% (2 SEs; *n* = 15).

In addition to the spot analyses, secondary ion maps were generated by rastering a ~5-nA Cs^+^ beam over areas of nominally 30 μm by 30 μm and sequentially detecting secondary ions of ^32^S^−^ and ^16^O_2_^−^ on a resistive anode encoder after an initial presputtering interval of ~180 s. Charge compensation of the raster area was again achieved by a normal incidence electron gun. A small (50 μm) contrast aperture was used to enhance lateral resolution to ~1 μm, which was determined from the 16 to 84% intensity change across a Si-Ta tuning grid using the ^30^Si^−^ signal. Ratios of ^32^S^−^ and ^16^O_2_^−^ were averaged pixel by pixel to obtain high-resolution qualitative S distribution maps.

### Imaging and microscopy

The polished section of HLK2 that was prepared for SIMS measurements was investigated using reflected light microscopy at the Institute of Earth Sciences, Heidelberg University, using a Keyence VHX-6000 digital microscope. In addition, confocal laser scanning fluorescence microscopy at the Heidelberg Nikon Imaging Center was performed using a Nikon AX Ti2 confocal microscope. Raster of confocal laser scanning fluorescence microscopy images was collected using an incident wavelength of 488 nm and a detection window of 499 to 551 nm with 10% overlap between individual fields. Depending on the desired level of detail, individual fields were acquired using Plan Apo λ 10× or 20× objectives [numerical aperture = 0.45 and 0.75, respectively] with a pinhole size of 30.1 μm. During acquisition of the images with a resolution of 2048 pixels by 2048 pixels, dwell times were set between 32 and 128 μs, and the intensities of two stacked frames were averaged to obtain a smoother image. Processing of confocal images including the extraction of fluorescence intensity profiles was performed using the open-source platform Fiji.

### Age distribution analyses

All age distributions are based on *n* = 10,000 Monte Carlo simulations. For the HLK2 speleothem, the LSE age distribution was calculated on the basis of the simulations performed to establish the speleothem chronology [i.e., the York fit age model (*55*)]. These simulations directly provide *n* ages for the LSE at the depth of the sulfur spike [i.e., 116.2 mm distance from top (dft)]. For the subfossil trees, the LSE age distribution is based on a normally distributed Monte Carlo simulation using the age and 2σ uncertainty of 13,006 ± 9 years B.P. (*9*). For the age distributions of the individual candidate events in the NGRIP ice core based on the U-Th–corrected GICC05 timescale (*23*), we assumed a triangular age distribution as suggested for the maximum counting error (MCE) of the layer-counted GICC05 chronology (*66*). Adolphi *et al.* (*23*) treated the MCE as an AR process and as ±1σ instead of ±2σ, which both leads to relatively large interpolation errors. Thus, their interpolation uncertainty is considered a conservative estimate. However, they stress that their procedure does not provide a realistic model of the ice-core layer counting process and its uncertainty. Therefore, to account for potential additional uncertainty, we used the 3σ uncertainty of their transfer function for the simulation of the triangular age distributions of the individual LSE candidate events.

The probability that two age distributions for the LSE (e.g., the HLK2 speleothem and the V6 candidate event at 12,980 years B.P.) are identical can then simply be calculated by integrating one age distribution (e.g., for HLK2) between the minimum and maximum age limits of the other age distribution (the V6 candidate event). As stated in the main text, this results in a probability of 12% for the V7 candidate event at 12,994 years B.P., 1% for the V6 candidate event, and <<1% for all other events. Thus, at the 99% confidence level and on the U-Th–corrected GICC05 timescale (*23*), V7 is the only candidate event that agrees in timing and uncertainty with the independent LSE age of the HLK2 speleothem.

Joint age distributions were calculated by conflation (*67*, *68*). Conflation is used for consolidating data from independent experiments (in our case, independent climate archives and proxy data) that are designed to measure the same quantity (in our case, the age of the LSE).

### Correlation analysis

The correlation analysis is also based on a Monte Carlo simulation of the individual chronologies within their corresponding uncertainties. For the HLK2 speleothem, the corresponding Monte Carlo simulations are directly provided by the York fit age model (*55*). For the NGRIP GICC05 timescale, which is based on annual layer counting, this is less straightforward. Our approach is based on simulating the MCE at the bottom of the GICC05 chronology (i.e., 2611 years at 2428.78 m dft corresponding to an age of 60,200 years before 2000 CE) using a triangular distribution (*66*). Since the MCE is calculated as the accumulated sum of the uncertain layers counted as 0.5 ± 0.5 years (*66*), the MCE of 2611 years corresponds to 5222 potentially uncertain layers. The distribution of these uncertain layers over the core is known and can be derived from the evolution of the MCE with depth. For each iteration and each simulation of the MCE at the bottom of the chronology, we then sample the corresponding number of uncertain layers from the total distribution of uncertain layers. This results in *n* = 10,000 consistent chronologies of the GICC05 timescale.

The distribution of the correlation coefficients between the HLK2 and NGRIP δ^18^O records (Fig. 3C) is then derived by calculating the correlation between the two records for each iteration and, thus, each simulation of the two timescales. All records were smoothed by kernel regression smoothing (*69*) using the “glkerns()” function from R-package “lokern” (*70*) with a bandwidth of 50 years. Because both chronologies are simulated simultaneously, the maximum of the simulated correlation coefficient does not correspond to a specific absolute age but rather to a specific age offset between the two records maximizing the correlation.

## Supplementary Materials

### The PDF file includes:

Supplementary Text Figs. S1 to S6 Legend for data S1 References
