# Supplementary material for: Discovery of Laacher See eruption in speleothem record synchronizes Greenland and central European Late Glacial climate change
Source: Sci Adv. 2025 Jan 15;11(3):eadt4057. doi: 10.1126/sciadv.adt4057 (PMC11734736; doi:10.1126/sciadv.adt4057)
Supplement: Supplementary file 1 — Supplementary Text Figs. S1 to S6 Legend for data S1 References [file sciadv.adt4057_sm.pdf]

Science Advances
AAAS

# Supplementary Materials for

## Discovery of Laacher See eruption in speleothem record synchronizes Greenland and central European Late Glacial climate change

Sophie F. Warken *et al.*

Corresponding author: Sophie F. Warken, sophie.warken@uni-heidelberg.de

*Sci. Adv.* **11**, eadt4057 (2025)
DOI: 10.1126/sciadv.adt4057

**The PDF file includes:**

Supplementary Text
Figs. S1 to S6
Legend for data S1
References

**Other Supplementary Material for this manuscript includes the following:**

Data S1

**Supplementary Text 1**

Speleothem chronology

Previous data demonstrate a late glacial growth of speleothem HLK2 starting from 13.62 ± 0.13 ka BP (*32*). Growth notably decreased and possibly even ceased intermittently, between 12 and 13 ka BP. In this study, the age-depth-relationship of this growth phase was refined by analyzing five additional $^{230}$Th/U dates at the Institute for Geosciences, JGU Mainz (Fig. S **1**). All ages are in stratigraphic order within uncertainty and the updated data improve the age-depth control of HLK2 by a factor of three, with uncertainties of the corrected ages of c. 50 years (table S1).

In addition, confocal laser scanning microscopy reveals that an annual fluorescent banding is visible in HLK2, and in particular before and after the LSE eruption (Fig. S **2**). Annual fluorescent laminae require annual fluxes of fluorescent material such as 'humic' substances from the overlying soil onto the stalagmite (*77, 78*). In the temperate regions of Europe, the seasonal peak of dissolved organic carbon and soil-derived organic substances occurs in autumn, after the summer period of soil moisture deficit (*78, 79*). Cave monitoring of Herbstlabyrinth confirms that the transport of organic matter in drip waters occurs on a seasonal scale (*53, 54*). Repeated counting of the fluorescent seasonal banding across several transects suggests that the section between 119.3 and 113.5 mm dft contains between 260 and 275 laminae. According to this counting, the mean annual growth rate is c. 25 µm/a and varies between c. 15 and 35 µm/a (Fig. S 4). This floating layer counting chronology thus suggests near linear growth in this section, and also provides a very precise age control relative to the LSE geochemical anomaly for both the ion probe sulphur and $\delta^{18}$O records, as well as the LA-ICP-MS line scan measurements which were performed on the same section.

The age-depth relationship for the late glacial growth phase of HLK2 was constructed using StalAge (*76*). Between c. 120 and c. 112 mm dft, the StalAge chronology shows a remarkable agreement with the floating layer counting, suggesting nearly linear growth. Therefore, we additionally applied a linear York fit model of the $^{230}$Th/U ages for this section (*55*). The MSWD of the fit is 1.05, and the corresponding Probability of Fit is 0.39. This confirms that the age data of the section between c. 120 and c. 112 mm dft satisfy the assumptions of the weighted linear fit [(*80*)]. According to this age model, the timing of the onset of the LSE peak at 116.2 ± 0.2 mm dft corresponds to 13.049 ± 30 years $BP_{1950}$ (95 %-confidence limit). Note that for the whole record, we use the StalAge age-depth-relationship (Figure 2).

Geochemical evidence of the LSE

At 116.2 mm dft, a distinct geochemical anomaly is recorded in speleothem HLK2. It is associated with an abrupt increase in the content of fluorescent material, a sharp peak in sulphur abundance, as well as higher concentrations of certain trace metals. In this paragraph, this geochemical anomaly associated with the Laacher See volcanic eruption is described and discussed in more detail.

Confocal laser scanning fluorescence microscopy reveals not only a seasonal banding, but also a distinct layer at 116.2 ± 0.2 mm dft which stands out with an abrupt increase in fluorescence intensity, which slowly declines in the following 100–200 µm (Fig. S **2**). This pronounced layer is indicative of a strong flux of organic material into the speleothem (*81-84*). This is interpreted to

result from the enhanced production, mobilization, and transport of degraded organic matter after the eruption (*14, 34, 85*). Both drip water analyses as well as the annual fluorescence lamination in HLK2 imply that a seasonal flux of organic matter takes place within a few weeks or months after the onset of the infiltration season (*53, 54, 77*). Hence, the transport and subsequent incorporation of ash-derived components into the speleothem occurred either directly after the eruption, or in the following autumn, when the infiltration season starts (*43*).

Conventional light microscopy reveals that the geochemical anomaly at 116.2 mm dft is not associated with a growth stop but occurs close to a change in the porosity of speleothem HLK2 (Fig. S **2**). In the bottom part, the material is characterized by high porosity and opaque, milky calcite. Around 117 mm dft the fabric changes to a more translucent structure with a low porosity. However, a detailed comparison of the light microscopy images and fluorescence microscopy with the geochemical results reveals that the transition in porosity occurs gradually, and in particular, it is laterally not homogeneous. In contrast, the LSE horizon appears to be a laterally very distinct and uniform layer, with abrupt increases in fluorescence and S abundances. In some sections, the porosity changes already about 500–1000 µm before the fluorescence and S spike (Fig. S **2**). Hence, we conclude that the change in porosity and the geochemical anomaly associated with the LSE are independent phenomena and not caused by the same process and/or event.

The most convincing evidence for a volcanic eruption is the abrupt peak in Sulphur abundances in speleothem HLK2 (*27, 30*). The occurrence, magnitude, and width of this peak are independently replicated using different analytical methods, in particular, ultra-high resolution ion probe (SIMS) measurements as well as LA-ICP-MS line scans at different transects across the layer of interest. Additional support comes from electron probe microanalysis (EPMA) as well as ion probe (SIMS) elemental abundance mapping, both performed at the Institute of Earth Sciences, Heidelberg University (Fig. S **2**). Altogether, these methods reveal a consistent, compelling picture of the S distribution in speleothem HLK2. The S content abruptly from a background level of c. 50 µg/g to values of 700–1000 µg/g at 116.2 mm dft (Fig. S 3-S 5). The partially visible saw-tooth pattern suggests that the incorporation occurred in an active lateral growth surface. After the initial peak to maximal values of c. 800 µg/g around 116.2 ± 0.2mm dft, the S content slowly decreases and remains at a generally higher level than compared to the pre-event concentrations (Fig. S **2**).

LA-ICP-MS analyses along the two transects across the LSE geochemical anomaly confirm that this layer is not only associated with a distinct enrichment of S, but also an increase by about a factor of 2–3 in the abundances of other elements such as Ba, U, or Na at 116.2 mm dft (fig. S 4). While Mg or Sr show no clear change across the LSE horizon, a small peak in P contents is visible. In particular the S and Na anomalies are very distinct within the analyzed area, in particular between 135 and 108 mm dft, which covers the whole late glacial growth phase of HLK2 (fig. S 5). The abrupt enrichment of Ba, U, and P is also substantial, and pronounced in particular for U and Ba. However, U and Ba concentrations are similarly high in the bottom part of the growth phase, as well as around 110 mm dft, which could be attributed to drier conditions during the early late glacial, as well as the Younger Dryas, as additionally indicated by relatively high Mg contents (fig. S 4, Mischel, Scholz, Spotl, Jochum, Schroder-Ritzrau and Fiedler (*32*)).

Sulfates and soluble salts containing various mobile and volatile metals are typical chemical compounds leached from pristine tephra (*86*). Various volcanogenic chemical compounds dissolve rapidly in surface environments after the direct wet deposition of acid rain from the eruption plume and/or after leaching by water from freshly deposited tephra (*28, 86, 87*). A change to a more

oxidizing environment in the soil due to a decrease in soil pH after the eruption may have additionally promoted the mobilization of water-soluble elements from volcanic glass (*28, 86, 87*). The increase in P content could reflect the increase of organic substances, as revealed by fluorescence microscopy (compare previous section). Metals such as U or Ba also sorb to organic colloids, which further facilitate the mobilization and transport of these elements through the karst zone (*88*). Lower soil pH after the eruption could have contributed to the mobilization of these colloidally-bound elements or have enhanced cation exchange with clay minerals in the soil (*89*). Other elements potentially leached from the LSE tephra (e.g., Sr or Rb) lack a clear signal in the speleothem calcite. We interpret this to reflect other transport/mobilization pathways, a potential incompatibility with the crystal lattice, e.g., due to the ion radii of the elements or their inability to replace $Ca^{2+}$in the lattice, and – in the case of Sr – a high background signal from the carbonate host rock. The absence of a clear volcanogenic peak in Sr could be related to the increased presence of Na which has been suggested to influence calcite growth and the incorporation of elements, such as Sr (*90, 91*).

In summary, the abrupt increase of S, Ba, U, or Na at 116.2 mm dft can be attributed to be derived from leaching of these elements either directly from the LSE tephra, or indirectly from the overlying soil via changes in soil pH. Collectively, these textural and geochemical anomalies are unique for the entire speleothem and reflect the dramatic environmental impact of the LSE in a proximal location for which ~20 cm thick tephra fallout is expected (*31*) (Figure 1 in main text).

Composite $\delta^{18}O$ record using ISCAM

The IRMS and SIMS stable oxygen isotope data of HLK2 were combined into a composite record using the software ISCAM (*65*). ISCAM was run with 50 AR1 simulations, and 100 Monte Carlo (MC) runs for each AR1. For the age distributions 1000 MC runs were implemented. The data was smoothed over 5 years before the correlation, but not detrended or normalized. Figure S4 shows the original records and the final composite against distance from the top.

Climatic interpretation of speleothem proxies at the BA/YD boundary

The stable oxygen isotope and trace element record of speleothem HLK2 allows inferences on climatic changes, in particular at the transition from the Bolling/Allerød into the Younger Dryas. This boundary is the most prominent feature of the record, with a pronounced drop of $\delta^{18}O$ values by c. 2 ‰ at c. 113-113.5 mm dft. Based on the modern sensitivity of $\delta^{18}O$ values of local precipitation to temperature (decrease in $\delta^{18}O$ precipitation values by c. 0.2‰ per decrease of 1 °C (*43*)) only about 40-60 % of the decrease in speleothem $\delta^{18}O$ values can be attributed to the c. 4–6 °C temperature drop related to the onset of the YD in western Europe (*92*). The remaining isotopic decrease may be related to a decline in North Atlantic surface water $\delta^{18}O$ values owing to the assumed freshwater release at that time (*5*). This is consistent with previous work, demonstrating that Herbstlabyrinth cave $\delta^{18}O$ values are closely linked to the North Atlantic moisture source from interannual to millennial timescales (*32, 41, 43, 48*). In addition, this is consistent with other studies showing that speleothem $\delta^{18}O$ values from central Germany reflect large-scale changes in the North Atlantic and (pan-) regional atmospheric conditions (e.g., (*49-51, 92-95*)). Synchronous to the drop in speleothem $\delta^{18}O$ values, we observe not only a decrease in annual growth rate, but also an increase in Mg/Ca ratios (Extended Data Figure 2) indicative of decreasing effective moisture (*32, 96*). Since

regional temperatures also decreased, this trend in Mg/Ca likely reflects a change in rainfall amount rather than increased evaporation. This clearly demonstrates that hydroclimatic changes in central Germany occurred synchronous to the cooling associated with the Younger Dryas.

**Supplementary Figs. S1 – S6**

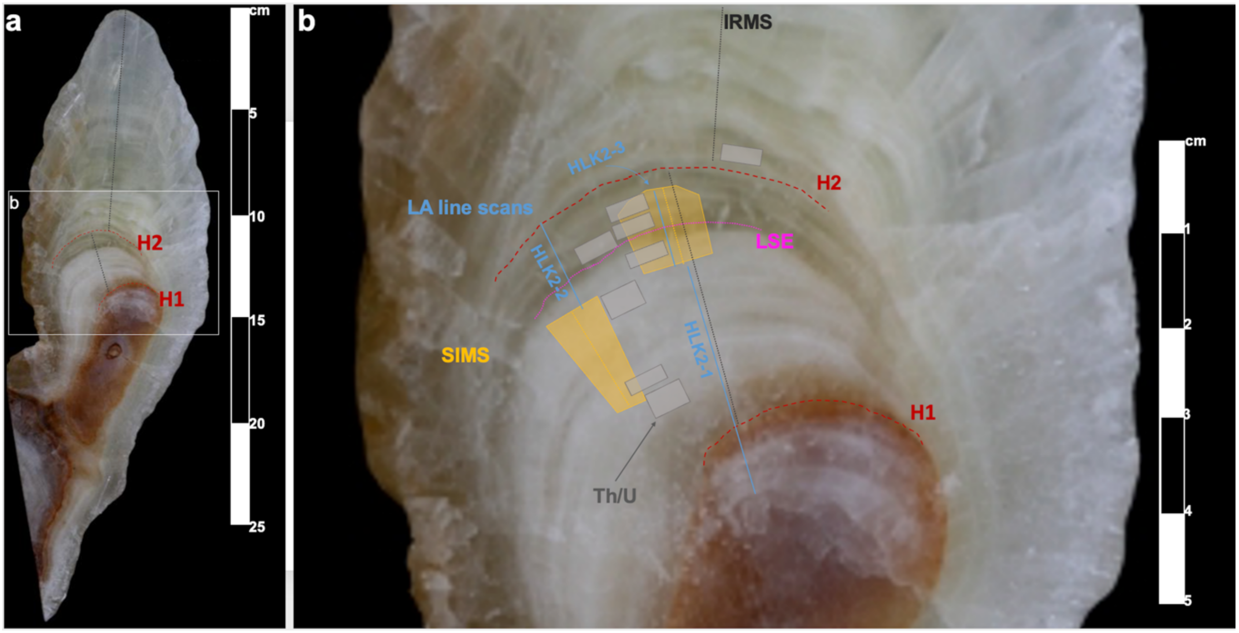


**Fig. S 1. Stalagmite HLK2 and sampling strategy.**

A: Total view of stalagmite HLK2 (modified after Mischel, Scholz, Spotl, Jochum, Schroder-Ritzrau and Fiedler (*32*)). Grey dashed line marks the central growth axis. B: Micro-sampling area within the late glacial growth phase. Grey dashed line indicates the track of the low resolution IRMS $\delta^{18}O$ analyses (*32*). The yellow shaded area depicts the sections that were prepared for SIMS analyses, while the yellow dashed lines indicate the sampling tracks for SIMS analyses, respectively. Blue lines indicate the line scans for elemental analysis using LA-ICP-MS. Grey rectangles show the sampling locations for $^{230}$Th/U age determinations. The red dashed line shows the location of the hiatuses H1 and H2, while the magenta dashed line indicates the LSE horizon at 116.2 mm dft.

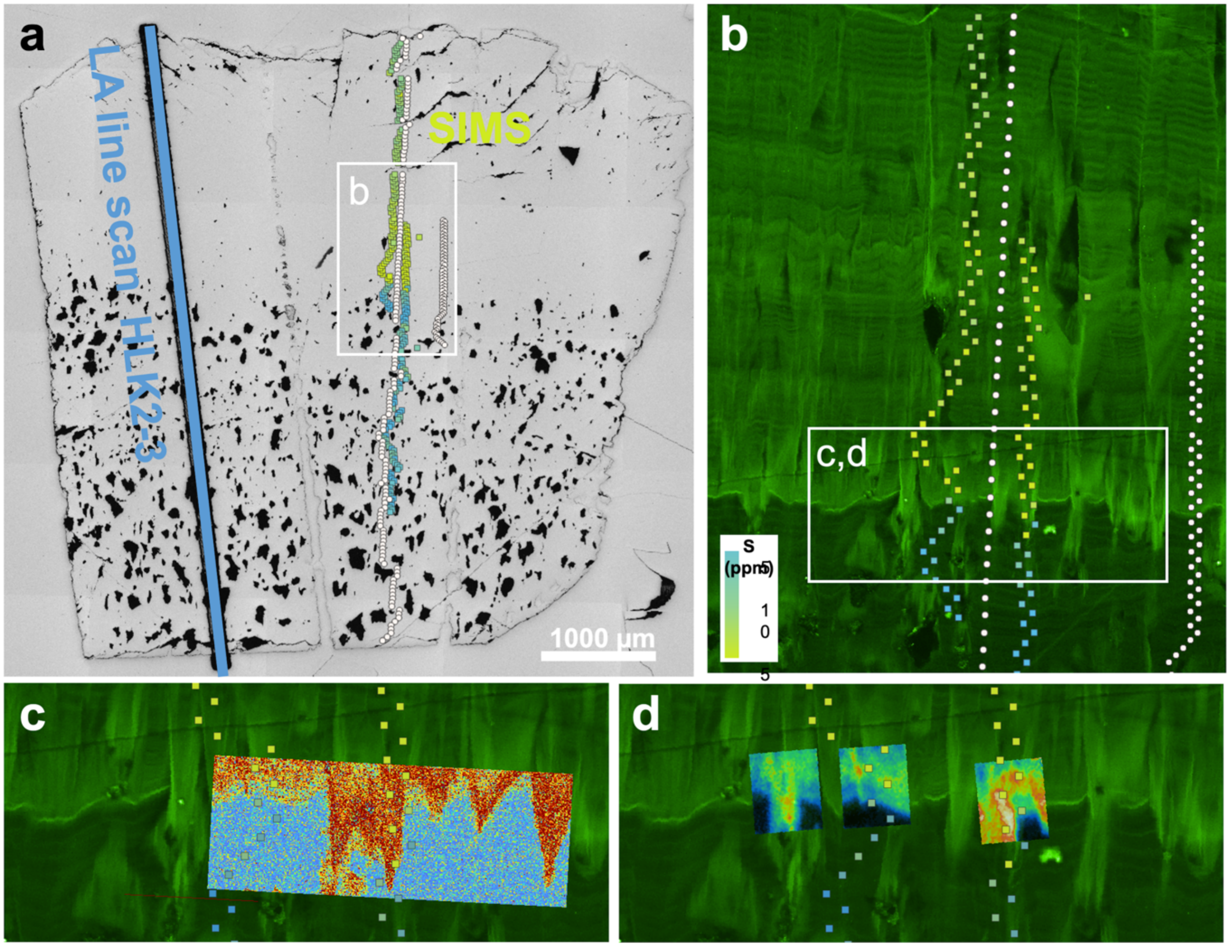


**Fig. S 2. Detailed view of HLK2 micro-sampling area around the LSE search window.**

(A) Reflected light microscope image of the polished mount used for SIMS and LA-ICP-MS analyses. (B)-(D) Confocal laser scanning fluorescent images of the LSE area. In addition, panel (B) shows the SIMS S (colored symbols) and $\delta^{18}O$ (white symbols) sampling points. Panel (C) and (D) compare SIMS S point measurements compared with EPMA (C) and ion probe (D) S abundance maps. In the maps, blue (red) colors indicate low (high) S abundances.

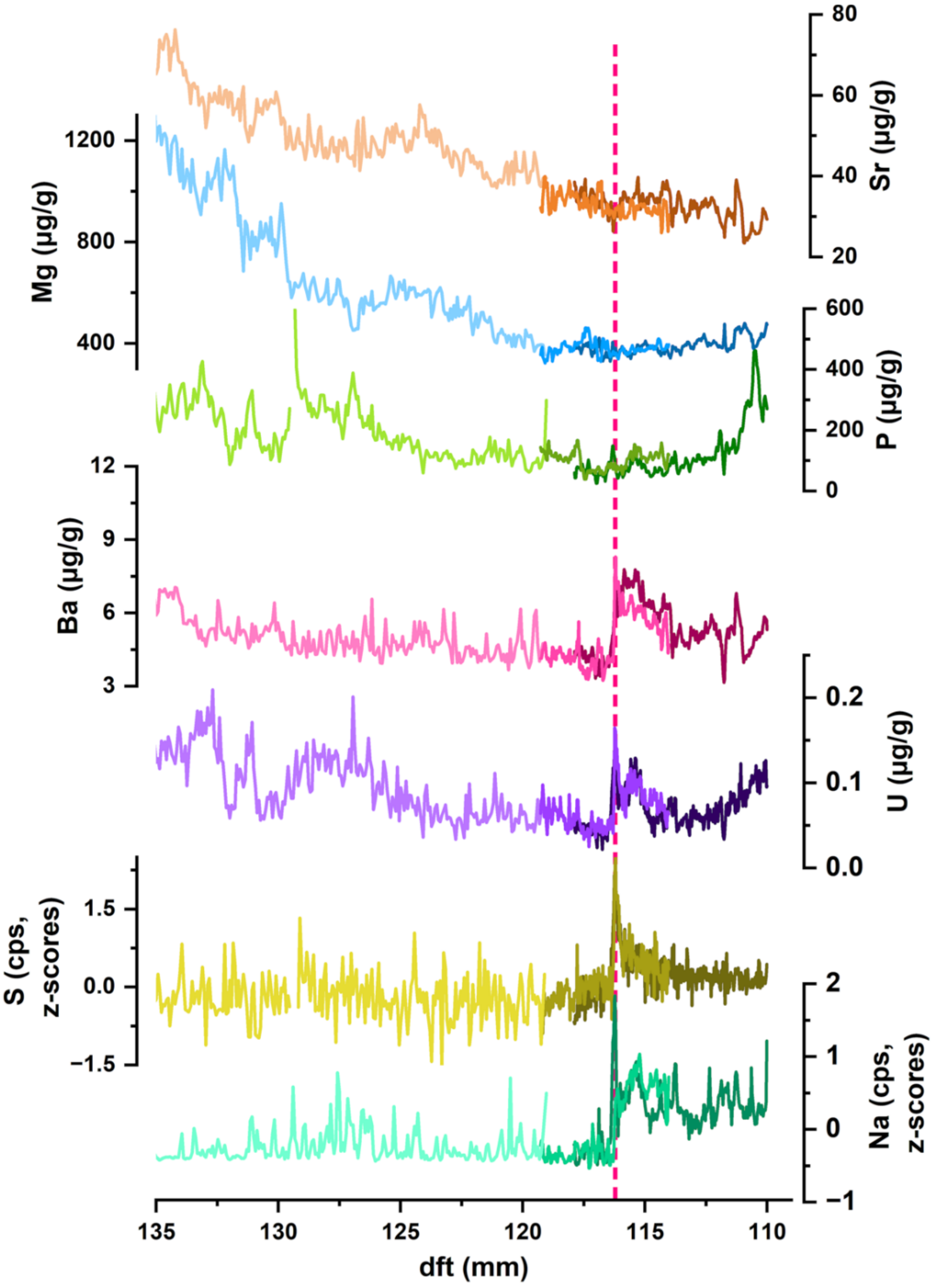


**Fig. S 3. LA-ICP-MS results of HLK2.**

LA-ICP-MS results for the linescans HLK2-1, HLK2-2, and HLK2-3 against distance from top downsampled to a resolution of 0.07, 0.03, and 0.03 mm (accounting for the different laser settings), respectively. Due to different laser settings of the different linescans and partially low signal-to-noise ratios, the elements S and Na are shown as normalized count rates. The LSE geochemical anomaly region is highlighted with a vertical dashed line at 116.2mm dft.

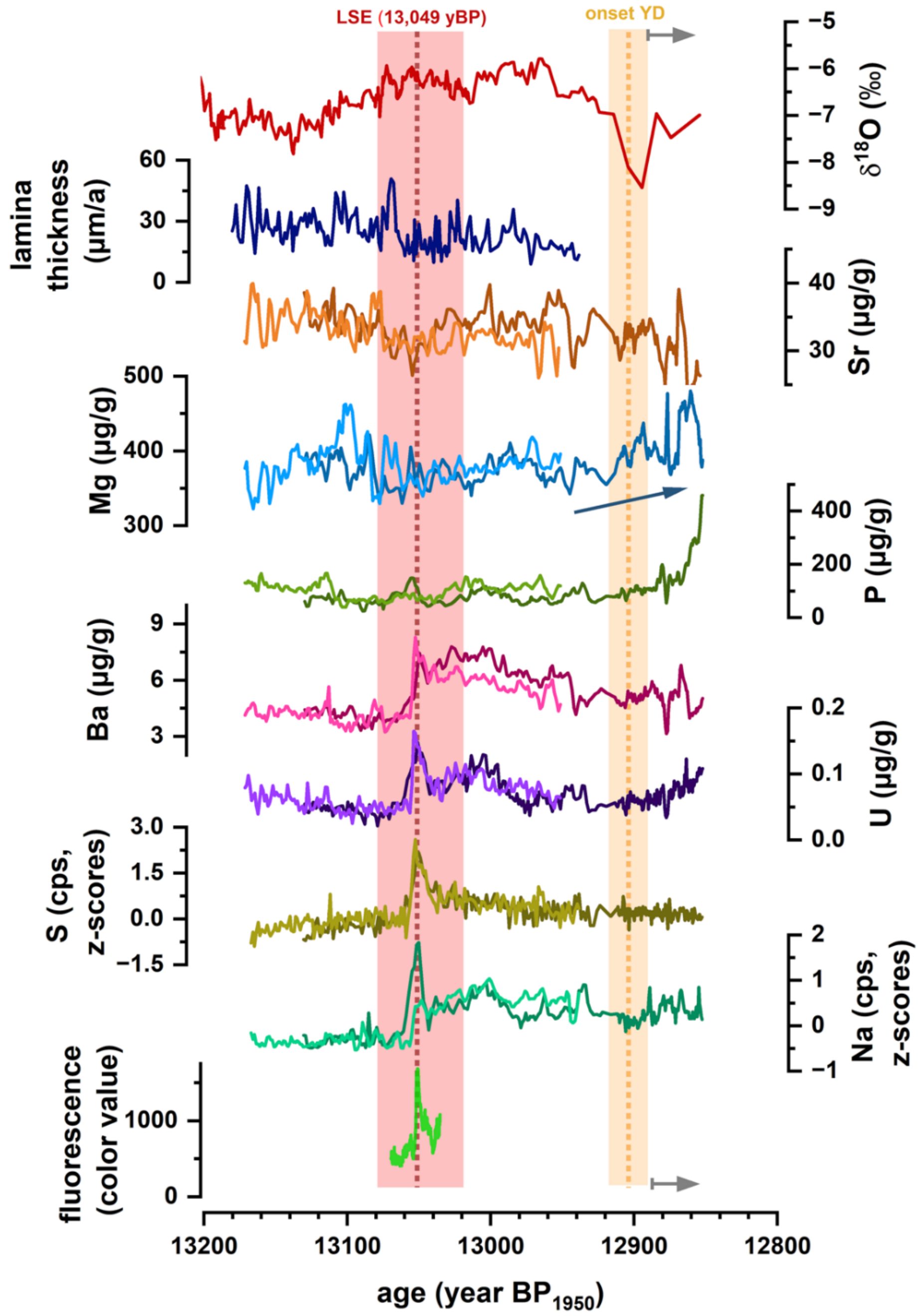


**Fig. S 4: Results of HLK2 $\delta^{18}O$ and trace element analyses.**

Lines indicate data from two coeval line scans (HLK2-2 and HLK2-3, compare **Fig. S 1**) vs. age on the $^{230}Th/U$ timescale down-sampled to equidistant resolution. The bottommost panel shows fluorescence intensity (green) obtained as color value from a transect parallel to HLK2-3 (compare **Fig. S 2**). The data covers the LSE geochemical anomaly (vertical red bar) until the onset of the Younger Dryas as indicated by orange dashed line and shading. Gray arrows indicate the section beyond 113mm dft where the chronology could not be constrained with sufficient accuracy (compare age model in main fig. 2).

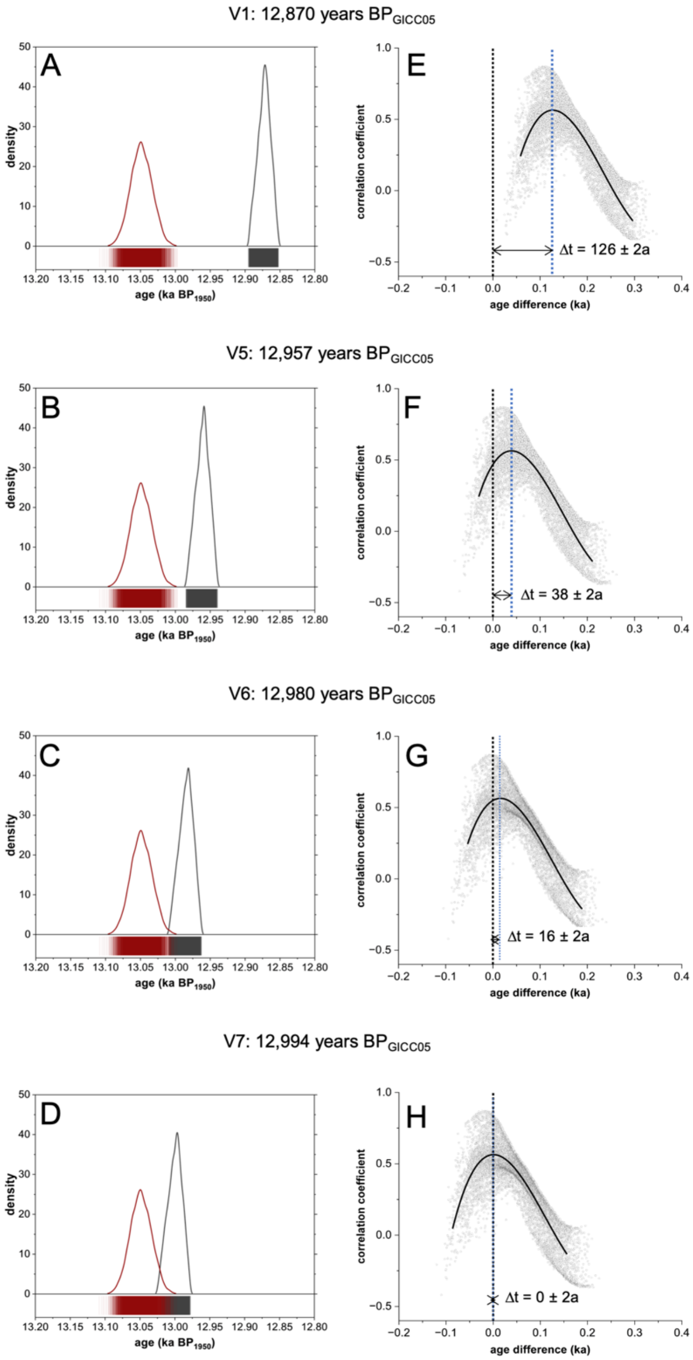


**Fig. S 5: Age distribution and correlation analysis.**

A-D) Age distribution density plots of LSE in HLK2 (red) and candidate events V1 and V5-7 in NGRIP. E-H) Correlation coefficients between speleothem HLK and NGRIP $\delta^{18}O$ records vs. the simulated age difference Δt for the candidate events V1 and V5-7. The black lines are 3rd order polynomial fits of the individual correlation coefficients (grey symbols). Only for V7 (12,994 BPGICC05, panel H) does the maximum of the correlation (dashed blue line) coincide with a simulated age difference of zero (dashed black line).

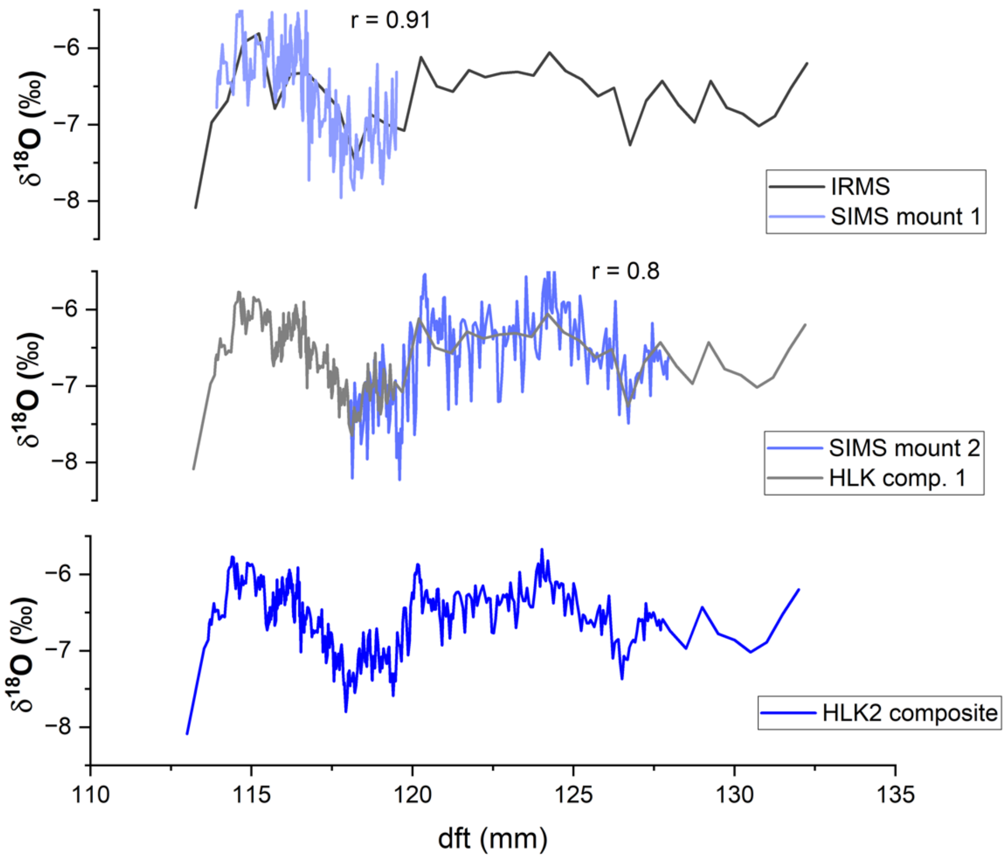


**Fig. S 6: HLK2 composite $\delta^{18}O$ record.**

Construction of the composite $\delta^{18}O$ record of speleothem HLK2 using ISCAM (*65*).

**Data S1. (separate file)**

The supplementary data file contains all data and information relevant to this study, including $^{230}$ThU ages of speleothem HLK2 (table S1), LA-ICP-MS results of speleothem HLK2 (table S2), SIMS S concentrations (table S3), SIMS O isotopes results (table S4), Composite O isotope record (table S5), and a list of the candidate events for the LSE in the ice core record (table S6).

## REFERENCES AND NOTES

1. V. Gkinis, M. Guillevic, W. Z. Hoek, J. John Lowe, J. B. Pedro, T. Popp, I. K. Seierstad, J. P. Steffensen, A. M. Svensson, P. Vallelonga, B. M. Vinther, M. J. C. Walker, J. J. Wheatley, M. Winstrup, A stratigraphic framework for abrupt climatic changes during the Last Glacial period based on three synchronized Greenland ice-core records: Refining and extending the INTIMATE event stratigraphy. *Quat. Sci. Rev.* **106**, 14–28 (2014).

2. R. B. Alley, The Younger Dryas cold interval as viewed from central Greenland. *Quat. Sci. Rev.* **19**, 213–226 (2000).

3. H. Cheng, H. Zhang, C. Spötl, J. Baker, A. Sinha, H. Li, M. Bartolomé, A. Moreno, G. Kathayat, J. Zhao, X. Dong, Y. Li, Y. Ning, X. Jia, B. Zong, Y. A. Brahim, C. Pérez-Mejías, Y. Cai, V. F. Novello, F. W. Cruz, J. P. Severinghaus, Z. An, R. L. Edwards, Timing and structure of the Younger Dryas event and its underlying climate dynamics. *Proc. Natl. Acad. Sci. U.S.A.* **117**, 23408–23417 (2020).

4. W. S. Broecker, J. P. Kennett, B. P. Flower, J. T. Teller, S. Trumbore, G. Bonani, W. Wolfli, Routing of meltwater from the Laurentide Ice Sheet during the Younger Dryas cold episode. *Nature* **341**, 318–321 (1989).

5. J. F. McManus, R. Francois, J. M. Gherardi, L. D. Keigwin, S. Brown-Leger, Collapse and rapid resumption of Atlantic meridional circulation linked to deglacial climate changes. *Nature* **428**, 834–837 (2004).

6. S. Rahmstorf, J. E. Box, G. Feulner, M. E. Mann, A. Robinson, S. Rutherford, E. J. Schaffernicht, Exceptional twentieth-century slowdown in Atlantic Ocean overturning circulation. *Nat. Clim. Change* **5**, 475–480 (2015).

7. R. M. van Westen, M. Kliphuis, H. A. Dijkstra, Physics-based early warning signal shows that AMOC is on tipping course. *Sci. Adv.* **10**, eadk1189 (2024).

8. C. S. Lane, A. Brauer, S. P. E. Blockley, P. Dulski, Volcanic ash reveals time-transgressive abrupt climate change during the Younger Dryas. *Geology* **41**, 1251–1254 (2013).

9. F. Reinig, L. Wacker, O. Jöris, C. Oppenheimer, G. Guidobaldi, D. Nievergelt, F. Adolphi, P. Cherubini, S. Engels, J. Esper, A. Land, C. Lane, H. Pfanz, S. Remmele, M. Sigl, A. Sookdeo, U. Büntgen, Precise date for the Laacher See eruption synchronizes the Younger Dryas. *Nature* **595**, 66–69 (2021).

10. A. Brauer, C. Endres, C. Günter, T. Litt, M. Stebich, J. F. Negendank, High resolution sediment and vegetation responses to Younger Dryas climate change in varved lake sediments from Meerfelder Maar, Germany. *Quat. Sci. Rev.* **18**, 321–329 (1999).

11. P. Van den Bogaard, 40Ar/39Ar ages of sanidine phenocrysts from Laacher See Tephra (12,900 yr BP): Chronostratigraphic and petrological significance. *Earth Planet. Sci. Lett.* **133**, 163–174 (1995).

12. U. J. van Raden, D. Colombaroli, A. Gilli, J. Schwander, S. M. Bernasconi, J. van Leeuwen, M. Leuenberger, U. Eicher, High-resolution late-glacial chronology for the Gerzensee lake record (Switzerland): $\delta^{18}$O correlation between a Gerzensee-stack and NGRIP. *Palaeogeogr. Palaeoclimatol. Palaeoecol.* **391**, 13–24 (2013).

13. T. Litt, H.-U. Schmincke, B. Kromer, Environmental response to climatic and volcanic events in central Europe during the Weichselian Lateglacial. *Quat. Sci. Rev.* **22**, 7–32 (2003).

14. H.-U. Schmincke, C. Park, E. Harms, Evolution and environmental impacts of the eruption of Laacher See Volcano (Germany) 12,900 a BP. *Quat. Int.* **61**, 61–72 (1999).

15. J. U. Baldini, R. J. Brown, N. Mawdsley, Evaluating the link between the sulfur-rich Laacher See volcanic eruption and the Younger Dryas climate anomaly. *Clim. Past* **14**, 969–990 (2018).

16. O. Rach, A. Brauer, H. Wilkes, D. Sachse, Delayed hydrological response to Greenland cooling at the onset of the Younger Dryas in western Europe. *Nat. Geosci.* **7**, 109–112 (2014).

17. C. Textor, P. Sachs, H.-F. Graf, T. Hansteen, The 12 900 years BP Laacher See eruption: Estimation of volatile yields and simulation of their fate in the plume. *Geol. Soc. Lond. Spec. Publ.* **213**, 307–328 (2003).

18. P. M. Abbott, U. Niemeier, C. Timmreck, F. Riede, J. R. McConnell, M. Severi, H. Fischer, A. Svensson, M. Toohey, F. Reinig, M. Sigl, Volcanic climate forcing preceding the inception of the Younger Dryas: Implications for tracing the Laacher See eruption. *Quat. Sci. Rev.* **274**, 107260 (2021).

19. J. U. L. Baldini, R. J. Brown, F. B. Wadsworth, A. R. Paine, J. W. Campbell, C. E. Green, N. Mawdsley, L. M. Baldini, Possible magmatic $CO_2$ influence on the Laacher See eruption date. *Nature* **619**, E1–E2 (2023).

20. A. G. Hogg, C. J. N. Wilson, D. J. Lowe, C. S. M. Turney, P. White, A. M. Lorrey, S. W. Manning, J. G. Palmer, S. Bury, J. Brown, J. Southon, F. Petchey, Wiggle-match radiocarbon dating of the Taupo eruption. *Nat. Commun.* **10**, 4669 (2019).

21. R. N. Holdaway, B. Duffy, B. Kennedy, Reply to 'Wiggle-match radiocarbon dating of the Taupo eruption'. *Nat. Commun.* **10**, 4668 (2019).

22. E. Cook, P. M. Abbott, N. J. G. Pearce, S. Mojtabavi, A. Svensson, A. J. Bourne, S. O. Rasmussen, I. K. Seierstad, B. M. Vinther, J. Harrison, E. Street, J. P. Steffensen, F. Wilhelms, S. M. Davies, Volcanism and the Greenland ice cores: A new tephrochronological framework for the last glacial-interglacial transition (LGIT) based on cryptotephra deposits in three ice cores. *Quat. Sci. Rev.* **292**, 107596 (2022).

23. F. Adolphi, C. B. Ramsey, T. Erhardt, R. Lawrence Edwards, H. Cheng, C. S. M. Turney, A. Cooper, A. Svensson, S. O. Rasmussen, H. Fischer, R. Muscheler, Connecting the Greenland ice-core and U/Th timescales via cosmogenic radionuclides: Testing the synchroneity of Dansgaard–Oeschger events. *Clim. Past* **14**, 1755–1781 (2018).

24. S. P. E. Blockley, A. J. Bourne, A. Brauer, S. M. Davies, M. Hardiman, P. R. Harding, C. S. Lane, A. MacLeod, I. P. Matthews, S. D. F. Pyne-O'Donnell, S. O. Rasmussen, S. Wulf, G. Zanchetta, Tephrochronology and the extended intimate (integration of ice-core, marine and terrestrial records) event stratigraphy 8–128 kab2k. *Quat. Sci. Rev.* **106**, 88–100 (2014).

25. F. A. Lechleitner, S. Amirnezhad-Mozhdehi, A. Columbu, L. Comas-Bru, I. Labuhn, C. Pérez-

Mejías, K. Rehfeld, The potential of speleothems from Western Europe as recorders of regional climate: A critical assessment of the SISAL database. *Quaternary* **1**, 30 (2018).

26. E. C. Corrick, R. N. Drysdale, J. C. Hellstrom, E. Capron, S. O. Rasmussen, X. Zhang, D. Fleitmann, I. Couchoud, E. Wolff, Synchronous timing of abrupt climate changes during the last glacial period. *Science* **369**, 963–969 (2020).

27. A. Borsato, S. Frisia, P. M. Wynn, I. J. Fairchild, R. Miorandi, Sulphate concentration in cave dripwater and speleothems: Long-term trends and overview of its significance as proxy for environmental processes and climate changes. *Quat. Sci. Rev.* **127**, 48–60 (2015).

28. B. Klaes, G. Wörner, K. Kremer, K. Simon, A. Kronz, D. Scholz, C. W. Mueller, C. Höschen, J. Struck, H. W. Arz, S. Thiele-Bruhn, D. Schimpf, R. Kilian, High-resolution stalagmite stratigraphy supports the Late Holocene tephrochronology of southernmost Patagonia. *Commun. Earth Environ.* **3**, 23 (2022).

29. S. Badertscher, A. Borsato, S. Frisia, H. Cheng, R. Edwards, O. Tüysüz, D. Fleitmann, Speleothems as sensitive recorders of volcanic eruptions–The Bronze Age Minoan eruption recorded in a stalagmite from Turkey. *Earth Planet. Sci. Lett.* **392**, 58–66 (2014).

30. S. Frisia, S. Badertscher, A. Borsato, J. Susini, O. M. Göktürk, H. Cheng, R. Edwards, J. Kramers, O. Tüysüz, D. Fleitmann, The use of stalagmite geochemistry to detect past volcanic eruptions and their environmental impacts. *PAGES News* **16**, 25–26 (2008).

31. I. Dorsten, D. Harries, Fund von Laacher-See-Bims im Herbstlabyrinth-Adventhöhlen-System (Hessen). *Jb. nass. Ver. Naturkde.* **127**, 131–136 (2006).

32. S. A. Mischel, D. Scholz, C. Spotl, K. P. Jochum, A. Schroder-Ritzrau, S. Fiedler, Holocene climate variability in Central Germany and a potential link to the polar North Atlantic: A replicated record from three coeval speleothems. *Holocene* **27**, 509–525 (2017).

33. G. Wörner, H.-U. Schmincke, Mineralogical and chemical zonation of the Laacher See tephra sequence (East Eifel, W. Germany). *J. Petrol.* **25**, 805–835 (1984).

34. S. Engels, B. van Geel, N. Buddelmeijer, A. Brauer, High-resolution palynological evidence for vegetation response to the Laacher See eruption from the varved record of Meerfelder Maar (Germany) and other central European records. *Rev. Palaeobot. Palynol.* **221**, 160–170 (2015).

35. I. Hajdas, A. Michczyński, Age-depth model of lake Soppensee (Switzerland) based on the high-resolution 14C chronology compared with varve chronology. *Radiocarbon* **52**, 1027–1040 (2010).

36. C. I. Blaga, G.-J. Reichart, A. F. Lotter, F. S. Anselmetti, J. S. Sinninghe Damsté, A $TEX_{86}$ lake record suggests simultaneous shifts in temperature in Central Europe and Greenland during the last deglaciation. *Geophys. Res. Lett.* **40**, 948–953 (2013).

37. K. K. Andersen, N. Azuma, J.-M. Barnola, M. Bigler, P. Biscaye, N. Caillon, J. Chappellaz, H. B. Clausen, D. Dahl-Jensen, H. Fischer, J. Flückiger, D. Fritzsche, Y. Fujii, K Goto-Azuma, K. Grønvold, N. S. Gundestrup, M. Hansson, C. Huber, C. S. Hvidberg, S. J. Johnsen, U. Jonsell, J. Jouzel, S. Kipfstuhl, A. Landais, M. Leuenberger, R. Lorrain, V. Masson-Delmotte, H. Miller, H. Motoyama, H. Narita, T. Popp, S. O. Rasmussen, D. Raynaud, R. Rothlisberger, U. Ruth, D. Samyn, J. Schwander, H. Shoji, M.-L. Siggard-Andersen, J. P. Steffensen, T. Stocker, A. E. Sveinbjörnsdóttir, A. Svensson, M. Takata, J.-L. Tison, T. Thorsteinsson, O. Watanabe, F. Wilhelms, J. W. C. White, North Greenland Ice Core Project members, High-resolution record of Northern Hemisphere climate extending into the last interglacial period. *Nature* **431**, 147–151 (2004).

38. C. B. Ramsey, P. G. Albert, S. P. Blockley, M. Hardiman, R. A. Housley, C. S. Lane, S. Lee, I. P. Matthews, V. C. Smith, J. J. Lowe, Improved age estimates for key Late Quaternary European tephra horizons in the RESET lattice. *Quat. Sci. Rev.* **118**, 18–32 (2015).

39. U. Niemeier, F. Riede, C. Timmreck, Simulation of ash clouds after a Laacher See-type eruption. *Clim. Past* **17**, 633–652 (2021).

40. G. Plunkett, J. R. Pilcher, Defining the potential source region of volcanic ash in northwest Europe during the Mid-to Late Holocene. *Earth Sci. Rev.* **179**, 20–37 (2018).

41. S. Waltgenbach, D. F. Riechelmann, C. Spötl, K. P. Jochum, J. Fohlmeister, A. Schröder-Ritzrau, D. Scholz, Climate variability in central Europe during the last 2500 years reconstructed from four high-resolution multi-proxy speleothem records. *Geosciences* **11**, 166 (2021).

42. J. L. Bernal-Wormull, A. Moreno, C. Pérez-Mejías, M. Bartolomé, A. Aranburu, M. Arriolabengoa, E. Iriarte, I. Cacho, C. Spötl, R. L. Edwards, H. Cheng, Immediate temperature response in northern Iberia to last deglacial changes in the North Atlantic. *Geology* **49**, 999–1003 (2021).

43. S. A. Mischel, D. Scholz, C. Spötl, $\delta^{18}O$ values of cave drip water: A promising proxy for the reconstruction of the North Atlantic Oscillation? *Climate Dynam.* **45**, 3035–3050 (2015).

44. J. P. Steffensen, K. K. Andersen, M. Bigler, H. B. Clausen, D. Dahl-Jensen, H. Fischer, K. Goto-Azuma, M. Hansson, S. J. Johnsen, J. Jouzel, V. Masson-Delmotte, T. Popp, S. O. Rasmussen, R. Röthlisberger, U. Ruth, B. Stauffer, M.-L. Siggaard-Andersen, Á. E. Sveinbjörnsdóttir, A. Svensson, J. W. C. White, High-resolution Greenland ice core data show abrupt climate change happens in few years. *Science* **321**, 680–684 (2008).

45. U. von Grafenstein, H. Erlenkeuser, A. Brauer, J. Jouzel, S. J. Johnsen, A mid-European decadal isotope-climate record from 15,500 to 5000 years BP. *Science* **284**, 1654–1657 (1999).

46. S. Lauterbach, A. Brauer, N. Andersen, D. L. Danielopol, P. Dulski, M. Hüls, K. Milecka, T. Namiotko, M. Obremska, U. Von Grafenstein, Declakes Participants, Environmental responses to Lateglacial climatic fluctuations recorded in the sediments of pre-Alpine Lake Mondsee (northeastern Alps). *J. Quat. Sci.* **26**, 253–267 (2011).

47. P. Ditlevsen, S. Ditlevsen, Warning of a forthcoming collapse of the Atlantic meridional overturning circulation. *Nat. Commun.* **14**, 4254 (2023).

48. S. Waltgenbach, D. Scholz, C. Spötl, D. F. Riechelmann, K. P. Jochum, J. Fohlmeister, A. Schröder-Ritzrau, Climate and structure of the 8.2 ka event reconstructed from three speleothems from Germany. *Global Planet. Change* **193**, 103266 (2020).

49. S. F. Breitenbach, B. Plessen, S. Waltgenbach, R. Tjallingii, J. Leonhardt, K. P. Jochum, H.

Meyer, B. Goswami, N. Marwan, D. Scholz, Holocene interaction of maritime and continental climate in Central Europe: New speleothem evidence from Central Germany. *Global Planet. Change* **176**, 144–161 (2019).

50. J. Fohlmeister, A. Schröder-Ritzrau, D. Scholz, C. Spötl, D. F. C. Riechelmann, M. Mudelsee, A. Wackerbarth, A. Gerdes, S. Riechelmann, A. Immenhauser, D. K. Richter, A. Mangini, Bunker Cave stalagmites: An archive for central European Holocene climate variability. *Clim. Past* **8**, 1751–1764 (2012).

51. M. Deininger, F. McDermott, M. Mudelsee, M. Werner, N. Frank, A. Mangini, Coherency of late Holocene European speleothem $\delta^{18}O$ records linked to North Atlantic Ocean circulation. *Climate Dynam.* **49**, 595–618 (2017).

52. H. Li, C. Spötl, H. Cheng, A high-resolution speleothem proxy record of the Late Glacial in the European Alps: Extending the NALPS19 record until the beginning of the Holocene. *J. Quat. Sci.* **36**, 29–39 (2021).

53. J. M. Bosle, S. A. Mischel, A.-L. Schulze, D. Scholz, T. Hoffmann, Quantification of low molecular weight fatty acids in cave drip water and speleothems using HPLC-ESI-IT/MS—Development and validation of a selective method. *Anal. Bioanal. Chem.* **406**, 3167–3177 (2014).

54. I. Heidke, D. Scholz, T. Hoffmann, Lignin oxidation products as a potential proxy for vegetation and environmental changes in speleothems and cave drip water – A first record from the Herbstlabyrinth, central Germany. *Clim. Past* **15**, 1025–1037 (2019).

55. J. Klose, M. Weber, D. Scholz, Precisely dated speleothem growth phases as a proxy for particularly warm climate conditions during MIS 3 in Central Europe. *Commun. Earth Environ.* **5**, 719 (2024).

56. S. R. Taylor, S. M. McLennan, *The Continental Crust: Its Composition and Evolution* (Blackwell Scientific Publications, 1985).

57. K. Ludwig, J. Paces, Uranium-series dating of pedogenic silica and carbonate, Crater Flat,

Nevada. *Geochim. Cosmochim. Acta* **66**, 487–506 (2002).

58. K. P. Jochum, U. Nohl, K. Herwig, E. Lammel, B. Stoll, A. W. Hofmann, GeoReM: A new geochemical database for reference materials and isotopic standards. *Geostand. Geoanal. Res.* **29**, 333–338 (2005).

59. K. P. Jochum, U. Weis, B. Stoll, D. Kuzmin, Q. Yang, I. Raczek, D. E. Jacob, A. Stracke, K. Birbaum, D. A. Frick, D. Günther, J. Enzweiler, Determination of reference values for NIST SRM 610–617 glasses following ISO guidelines. *Geostand. Geoanal. Res.* **35**, 397–429 (2011).

60. C. Paton, J. Hellstrom, B. Paul, J. Woodhead, J. Hergt, Iolite: Freeware for the visualisation and processing of mass spectrometric data. *J. Anal. At. Spectrom* **26**, 2508–2518 (2011).

61. D. Garbe-Schönberg, P. C. Webb, P. J. Potts, S. Müller, J. Woodhead, "G-Probe 25b—An International Proficiency Test for Microanalytical Laboratories—Report on Round 25b (Speleothem, KCSp-1NP, Nano-particulate powder pellet)/December 2021" (International Association of Geoanalysts, 2021).

62. K. P. Jochum, D. Scholz, B. Stoll, U. Weis, S. A. Wilson, Q. Yang, A. Schwalb, N. Börner, D. E. Jacob, M. O. Andreae, Accurate trace element analysis of speleothems and biogenic calcium carbonates by LA-ICP-MS. *Chem. Geol.* **318-319**, 31–44 (2012).

63. P. Treble, A. K. Schmitt, R. Edwards, K. D. McKeegan, T. Harrison, M. Grove, H. Cheng, Y. Wang, High resolution secondary ionisation mass spectrometry (SIMS) $\delta^{18}O$ analyses of Hulu Cave speleothem at the time of Heinrich Event 1. *Chem. Geol.* **238**, 197–212 (2007).

64. R. A. Stern, CCIM-SIMS microbeam reference material chemistry: Calcite S0161, data release 06-2024 (2024).

65. J. Fohlmeister, A statistical approach to construct composite climate records of dated archives. *Quat. Geochronol.* **14**, 48–56 (2012).

66. K. K. Andersen, A. Svensson, S. J. Johnsen, S. O. Rasmussen, M. Bigler, R. Röthlisberger, U. Ruth, M.-L. Siggaard-Andersen, J. P. Steffensen, D. Dahl-Jensen, The Greenland ice core

chronology 2005, 15–42 ka. Part 1: Constructing the time scale. *Quat. Sci. Rev.* **25**, 3246–3257 (2006).

67. T. P. Hill, J. Miller, How to combine independent data sets for the same quantity. *Chaos* **21**, 033102 (2011).

68. T. Hill, Conflations of probability distributions. *Trans. Am. Math. Soc.* **363**, 3351–3372 (2011).

69. E. Herrmann, Local bandwidth choice in kernel regression estimation. *J. Comput. Graph. Stat.* **6**, 35–54 (1997).

70. E. Herrmann, M. M. Maechler, Package 'lokern' (2016).

71. F. Riede, O. Bazely, A. J. Newton, C. S. Lane, A Laacher See-eruption supplement to Tephrabase: Investigating distal tephra fallout dynamics. *Quat. Int.* **246**, 134–144 (2011).

72. S. Wulf, F. Ott, M. Słowiński, A. M. Noryśkiewicz, N. Dräger, C. Martin-Puertas, M. Czymzik, I. Neugebauer, P. Dulski, A. J. Bourne, M. Błaszkiewicz, A. Brauer, Tracing the Laacher See Tephra in the varved sediment record of the Trzechowskie palaeolake in central Northern Poland. *Quat. Sci. Rev.* **76**, 129–139 (2013).

73. G. Kletetschka, D. Vondrák, J. Hruba, W. O. van der Knaap, J. F. van Leeuwen, M. Heurich, Laacher See tephra discovered in the Bohemian Forest, Germany, east of the eruption. *Quat. Geochronol.* **51**, 130–139 (2019).

74. V. Procházka, J. Mizera, G. Kletetschka, D. Vondrák, Late Glacial sediments of the Stará Jímka paleolake and the first finding of Laacher See Tephra in the Czech Republic. *Int. J. Earth Sci.* **108**, 357–378 (2019).

75. S. Krüger, C. van den Bogaard, Small shards and long distances—Three cryptotephra layers from the Nahe palaeolake including the first discovery of Laacher See Tephra in Schleswig-Holstein (Germany). *J. Quat. Sci.* **36**, 8–19 (2021).

76. D. Scholz, D. L. Hoffmann, StalAge – An algorithm designed for construction of speleothem

age models. *Quat. Geochronol.* **6**, 369–382 (2011).

77. A. Baker, G. Mariethoz, L. Comas-Bru, A. Hartmann, S. Frisia, A. Borsato, P. C. Treble, A. Asrat, The properties of annually laminated stalagmites-A global synthesis. *Rev. Geophys.* **59**, e2020RG000722 (2021).

78. D. F. Riechelmann, J. Fohlmeister, T. Kluge, K. P. Jochum, D. K. Richter, M. Deininger, R. Friedrich, N. Frank, D. Scholz, Evaluating the potential of tree-ring methodology for cross-dating of three annually laminated stalagmites from Zoolithencave (SE Germany). *Quat. Geochronol.* **52**, 37–50 (2019).

79. C. J. Proctor, A. Baker, W. L. Barnes, R. A. Gilmour, A thousand year speleothem proxy record of North Atlantic climate from Scotland. *Climate Dynam.* **16**, 815–820 (2000).

80. K. Ludwig, Mathematical–statistical treatment of data and errors for $^{230}$Th/U geochronology. *Rev. Mineral. Geochem.* **52**, 631–656 (2003).

81. S. F. McGarry, A. Baker, Organic acid fluorescence: Applications to speleothem palaeoenvironmental reconstruction. *Quat. Sci. Rev.* **19**, 1087–1101 (2000).

82. A. J. Blyth, A. Baker, M. J. Collins, K. E. H. Penkman, M. A. Gilmour, J. S. Moss, D. Genty, R. N. Drysdale, Molecular organic matter in speleothems and its potential as an environmental proxy. *Quat. Sci. Rev.* **27**, 905–921 (2008).

83. A. J. Blyth, A. Hartland, A. Baker, Organic proxies in speleothems–New developments, advantages and limitations. *Quat. Sci. Rev.* **149**, 1–17 (2016).

84. M. Quiers, Y. Perrette, E. Chalmin, B. Fanget, J. Poulenard, Geochemical mapping of organic carbon in stalagmites using liquid-phase and solid-phase fluorescence. *Chem. Geol.* **411**, 240–247 (2015).

85. I. Obreht, L. Wörmer, A. Brauer, J. Wendt, S. Alfken, D. De Vleeschouwer, M. Elvert, K.-U. Hinrichs, An annually resolved record of Western European vegetation response to Younger Dryas cooling. *Quat. Sci. Rev.* **231**, 106198 (2020).

86. P. M. Ayris, P. Delmelle, The immediate environmental effects of tephra emission. *Bull. Volcanol.* **74**, 1905–1936 (2012).

87. M. T. Jones, S. R. Gislason, Rapid releases of metal salts and nutrients following the deposition of volcanic ash into aqueous environments. *Geochim. Cosmochim. Acta* **72**, 3661–3680 (2008).

88. J. Hellstrom, M. McCulloch, Multi-proxy constraints on the climatic significance of trace element records from a New Zealand speleothem. *Earth Planet. Sci. Lett.* **179**, 287–297 (2000).

89. C. V. Tadros, P. C. Treble, A. Baker, S. Hankin, R. Roach, Cave drip water solutes in south-eastern Australia: Constraining sources, sinks and processes. *Sci. Total Environ.* **651**, 2175–2186 (2019).

90. J. T. Sliwinski, H. M. Stoll, Combined fluorescence imaging and LA-ICP-MS trace element mapping of stalagmites: Microfabric identification and interpretation. *Chem. Geol.* **581**, 120397 (2021).

91. S. Frisia, A. Borsato, A. Hartland, M. Faraji, A. Demeny, R. N. Drysdale, C. E. Marjo, Crystallization pathways, fabrics and the capture of climate proxies in speleothems: Examples from the tropics. *Quat. Sci. Rev.* **297**, 107833 (2022).

92. S. Affolter, A. Häuselmann, D. Fleitmann, R. L. Edwards, H. Cheng, M. Leuenberger, Central Europe temperature constrained by speleothem fluid inclusion water isotopes over the past 14,000 years. *Sci. Adv.* **5**, eaav3809 (2019).

93. F. McDermott, T. C. Atkinson, I. J. Fairchild, L. M. Baldini, D. P. Mattey, A first evaluation of the spatial gradients in $\delta^{18}O$ recorded by European Holocene speleothems. *Global Planet. Change* **79**, 275–287 (2011).

94. S. Niggemann, A. Mangini, M. Mudelsee, D. K. Richter, G. Wurth, Sub-Milankovitch climatic cycles in Holocene stalagmites from Sauerland, Germany. *Earth Planet. Sci. Lett.* **216**, 539–547 (2003).

95. S. Riechelmann, A. Schröder-Ritzrau, C. Spötl, D. F. C. Riechelmann, D. K. Richter, A.

Mangini, N. Frank, S. F. M. Breitenbach, A. Immenhauser, Sensitivity of Bunker Cave to climatic forcings highlighted through multi-annual monitoring of rain-, soil-, and dripwaters. *Chem. Geol.* **449**, 194–205 (2017).

96. S. F. Warken, J. Fohlmeister, A. Schröder-Ritzrau, S. Constantin, C. Spotl, A. Gerdes, J. Esper, N. Frank, J. Arps, M. Terente, D. F. C. Riechelmann, A. Mangini, D. Scholz, Reconstruction of late Holocene autumn/winter precipitation variability in SW Romania from a high-resolution speleothem trace element record. *Earth Planet. Sci. Lett.* **499**, 122–133 (2018).
